# Displayed correlation between gene expression profiles and submicroscopic alterations in response to cetuximab, gefitinib and EGF in human colon cancer cell lines

**DOI:** 10.1186/1471-2407-8-227

**Published:** 2008-08-08

**Authors:** Rossella Solmi, Mattia Lauriola, Mirko Francesconi, Désirée Martini, Manuela Voltattorni, Claudio Ceccarelli, Giampaolo Ugolini, Giancarlo Rosati, Simone Zanotti, Isacco Montroni, Gabriella Mattei, Mario Taffurelli, Donatella Santini, Furio Pezzetti, Alessandro Ruggeri, Gastone Castellani, Lia Guidotti, Domenico Coppola, Pierluigi Strippoli

**Affiliations:** 1 Dipartimento di Istologia, Embriologia e Biologia Applicata, Università di Bologna, Via Belmeloro 8, 40126 Bologna, Italy; 2 Centro Interdipartimentale "L. Galvani", Università di Bologna, Bologna, Italy; 3 DIMORFIPA, Università di Bologna, Bologna, Italy; 4 Dipartimento di Scienze Anatomiche Umane e Fisiopatologia dell'Apparato Locomotore, Università di Bologna, Bologna, Italy; 5 Laboratori di Biotecnologie, Via Beverara 123, Bologna, Italy; 6 Dipartimento di Patologia, Università di Bologna, Bologna, Italy; 7 Dipartimento Emergenza/Urgenza, Chirurgia Generale e dei Trapianti, Università di Bologna, Bologna, Italy; 8 "H. Lee Moffit" Cancer Center and Research Institute, University of South Florida, Tampa, FL, USA

## Abstract

**Background:**

EGFR is frequently overexpressed in colon cancer. We characterized HT-29 and Caco-2, human colon cancer cell lines, untreated and treated with cetuximab or gefitinib alone and in combination with EGF.

**Methods:**

Cell growth was determined using a variation on the MTT assay. Cell-cycle analysis was conducted by flow cytometry. Immunohistochemistry was performed to evaluate EGFR expression and scanning electron microscopy (SEM) evidenced the ultrastructural morphology. Gene expression profiling was performed using hybridization of the microarray Ocimum Pan Human 40 K array A.

**Results:**

Caco-2 and HT-29 were respectively 66.25 and 59.24 % in G0/G1. They maintained this level of cell cycle distribution after treatment, suggesting a predominantly differentiated state. Treatment of Caco-2 with EGF or the two EGFR inhibitors produced a significant reduction in their viability. SEM clearly showed morphological cellular transformations in the direction of cellular death in both cell lines treated with EGFR inhibitors. HT-29 and Caco-2 displayed an important reduction of the microvilli (which also lose their erect position in Caco-2), possibly invalidating microvilli absorption function. HT-29 treated with cetuximab lost their boundary contacts and showed filipodi; when treated with gefitinib, they showed some vesicles: generally membrane reshaping is evident. Both cell lines showed a similar behavior in terms of on/off switched genes upon treatment with cetuximab. The gefitinib global gene expression pattern was different for the 2 cell lines; gefitinib treatment induced more changes, but directly correlated with EGF treatment.

In cetuximab or gefitinib plus EGF treatments there was possible summation of the morphological effects: cells seemed more weakly affected by the transformation towards apoptosis. The genes appeared to be less stimulated than for single drug cases.

**Conclusion:**

This is the first study to have systematically investigated the effect of cetuximab or gefitinib, alone and in combination with EGF, on human colon cancer cell lines. The EGFR inhibitors have a weaker effect in the presence of EGF that binds EGFR. Cetuximab treatment showed an expression pattern that inversely correlates with EGF treatment. We found interesting cyto-morphological features closely relating to gene expression profile. Both drugs have an effect on differentiation towards cellular death.

## Background

Epidermal growth factor receptor (EGFR) is one of the most important cell membrane receptors expressed in normal cells [1]. The EGFR molecular structure, common to the other three members (ErbB2 or HER2/neu, ErbB3, ErbB4) of the ErbB receptor [2] family, includes an extracellular region, a transmembrane domain and a protein tyrosine kinase region [3]. Tyrosine kinase phosphorylation controls the intracellular signal transduction pathways regulating cell proliferation and differentiation [4]. Epidermal growth factor (EGF) is a natural ligand of EGFR. EGF binding to the EGFR ectodomain creates prolonged and stabilized conformation and sets about signaling with the dimerization of EGFR molecules or heterodimerization with other closely related receptors, such as HER2/neu [5].

EGFR is abnormally activated in many epithelial tumors and is frequently overexpressed in colon cancer correlating with poor response to treatment, disease progression, and poor survival [6].

In the early 1980s the EGFR pathway was pointed to as a potential target for cancer therapy [7,8] and two anti-EGFR strategies were adopted: monoclonal antibodies (Mabs) which bind the extracellular domain, interfering with the natural ligand, and low-molecular-weight tyrosine kinase inhibitors (TKIs) which interfere with ATP for the tyrosine kinase domain [9].

Cetuximab, a chimeric Mab, is a competitive antagonist for EGFR. Cetuximab binds to EGFR with high affinity and prevents the ligand from interacting with the receptor and the receptor from adopting the conformation required for dimerization [10-13]. Cetuximab may promote receptor internalization and degradation [14-16], although this does not happen in all systems. The mechanisms of the cetuximab-receptor complex degradation and of cell membrane recycling of the intact receptor are not clearly documented [15,17].

Moreover, cetuximab may elicit antibody-dependent cellular cytotoxicity (ADCC), a mechanism of cell-mediated immunity resulting in lysis of the target cells [18,19].

Gefitinib acts on the cytosolic ATP binding domain of EGFR by inhibiting EGFR autophosphorylation [20] but it is not strictly specific for EGFR [21] and some cross-reactivity is possible between EGFR and other HER-B family members [22]. Low-molecular-weight EGFR tyrosine kinase inhibitors induce formation of inactive EGFR homodimers and EGFR/HER2 heterodimers [23] which impair EGFR-mediated transactivation of HER2 tyrosine kinase [24-26].

These two types of agent have shown solid preclinical and clinical activity in a variety of tumor types [27]; the clinical data suggest they have different activity profiles [28,29].

For the experimental model of our study we chose two human colon cancer cell lines, HT-29 and Caco-2. These enterocyte cell lines were derived from two human primary colon adenocarcinomas and are well established models for the study of biology and drug treatment of colon cancer [30-33]. We characterized them as having high and moderate EGFR expression levels, respectively (as previously shown by other authors [34]) with a view to comparing their biological behavior after drug treatment. HT-29 are smaller than CaCo-2 and are more isolated than Caco-2 which form a very crowded confluence. The huge numbers of microvilli present in both cell lines are shorter in HT-29 than in Caco-2.

These two cell lines were treated with gefitinib, cetuximab and EGF. We also treated Caco-2 and HT-29 with gefitinib plus EGF and cetuximab plus EGF. In fact the natural ligand may compete with the binding of cetuximab to the receptor target or it may confer more dependency on the targeted cell through activation of the EGFR pathway and thus favor the activity of gefitinib [34,35].

The present work aims to compare the key factors governing the action of these three agents (cetuximab, gefitinib and EGF) on cell morphology and proliferation of Caco-2 and HT-29 cells. We also used cDNA arrays to analyze the changes in gene expression profiles induced by these agents. Our work shows interesting cyto-morphologic features possibly correlated to the clinical effects of cetuximab and gefitinib, which suggests that both drugs have an inhibiting effect and induce extreme cell differentiation towards cellular death. Cetuximab has opposite effects on gene expression profiling compared to EGF alone or gefitinib, indicating a different action mechanism than the other drug, even though the cell cyto-morphological transformations are sometimes the same, possibly suggesting an important role by translational regulation on the cellular pathways.

## Methods

### Compounds

EGFR-tyrosine kinase inhibitor gefitinib (ZD1839; Iressa; kindly provided by AstraZeneca Pharmaceuticals, Macclesfield, United Kingdom), monoclonal antibody anti-EGFR cetuximab (IMC-C225; Erbitux; kindly provided by Merck KGaA, Darmstadt, Germany), and Epidermal Growth Factor (EGF) purchased from SIGMA Saint Louis, Missouri, USA were used for *in vitro *assays.

### Cell Lines

HT-29 is a cell line isolated in this case from a primary colon adenocarcinoma grade II in a 44 year-old Caucasian female (60th to 65th passage), while Caco-2 was isolated from a primary colon adenocarcinoma in a 72 year-old Caucasian male (43rd to 50th passage). These human enterocyte lines were purchased from American Type Culture Collection (ATCC) and cultured in Dulbecco's minimal essential medium (DMEM), 25 mM glucose supplemented with 2 mM L-glutamine, antibiotics (100 U.mL^-1 ^penicillin and 100 mg.mL^-1 ^streptomycin) and with 10% (v/v) heat-inactivated fetal bovine serum (Cambrex, Verviers, Belgium). Cells were grown in a 37°C and 5% CO_2_/air incubator and the medium was changed every 3 days. For all experiments cells were treated at a 70–80% degree of confluency.

### Cell-viability assay

Cell growth was determined using a variation on the MTT [3-(4,5-dimethylthiazol-2-yl)-2,5-diphenyl tetrazolium bromide] assay described by Mosmann [36]. HT-29 and Caco-2 cells were counted using Trypan Blue solution 10 % in a Neubauer cell counter chamber (Brand, Wertheim, Germany) and observing viable (nonstained) and nonviable (stained) cells under a microscope [37]. Cells were seeded into 25 cm^2 ^tissue culture flasks (Becton Dickinson Labware Europe Le Pont De Claix, France) at 4.0 × 10^5 ^cells per flask and incubated for 5 days. After cells had been serum-starved for 24 h, EGF, gefitinib, cetuximab, EGF and gefitinib, EGF and cetuximab, were added at the concentrations indicated and the flasks were incubated for 24 h at 37°C. In order to establish the initial number of cells treated, extra flasks of Caco-2 and of HT-29 cells were treated with trypsin and then the cells were counted. The concentrations were: 10 nM EGF (the most frequent concentration used in the literature), 1 μmol/L gefitinib (recommended concentration by Astra Zeneca), 5 and 10 nmol/L cetuximab (recommended concentration by Merck), 1 μmol/L gefitinib plus 10 nM EGF, 5 and 10 nmol/L cetuximab plus 10 nM EGF. After drug incubation, cells were washed once with Phosphate Buffer Saline (PBS), harvested in 0.1% trypsin-1 mmol/L EDTA in PBS, and counted. Four independent experiments and four replicates for untreated and treated cells, respectively, were conducted.

### Cell-cycle analysis

Cell-cycle analysis was performed by flow cytometry. HT-29 and Caco-2 cells were treated in the same manner as the cell viability assay. After detachment they were washed twice with PBS and then resuspended in a solution containing 0.1 % sodium citrate, 0.1 % Nonidet 40, 50 μg/mL propidium iodide and 10 μg/mL RNAase. Cells were incubated for 30' at 37°C in the dark.

The cell cycle profiles were determined using a Biorad Bryte HS flow cytometry system [38] (Biorad, UK) and analyzed by Modfit software [38]. Four independent experiments and four replicates were conducted for untreated and treated cells, respectively.

### Immunohistochemistry

HT-29 and Caco-2 cells were seeded into Lab-Tek two chamber glass slides (Nunc, Naperville IL) at 8 × 10^4 ^cells per chamber and incubated for 5 days. The cells were then treated as per the cell viability assay. They were fixed in cold methanol for 10 min at -20°C. Fixed cells were dried for 3–5 min under laminar flow and then kept at -20°C until staining. Immunohistochemistry was performed using a non-biotin amplified method (Novolink, Novocastra Laboratories, Newcastle UK).

Slides were thawed for 1 min at room temperature (RT) and immersed in a 0.5% methanol/H_2_O_2 _solution for 10 min to abolish endogenous peroxidase activity, washed 3 times in distilled water and immersed in a PBS pH 7.2–7.4 solution for 10 min. Cells were incubated overnight at RT in a humidified atmosphere using an anti-EGFR monoclonal antibody (clone 31G7, Zymed Laboratories, CA, USA) diluted 1:120. Cells were washed in PBS and processed using the Novolink system according to the manufacturer's suggested procedure. The reaction was developed using a 3-3'-diaminobenzidine tetrahydrochloride 50 mg/100 ml PBS solution activated with hydrogen peroxide for 10 minutes. Cell nuclei were counterstained using Mayer's Hematoxylin, dehydrated to xylene and mounted with BioMount (Bio-Optica, Milan, Italy). Two independent experiments and four replicates for untreated and treated cells were conducted per experiment.

### Semiquantitative evaluation of EGFR immunostaining

EGFR membranous and cytoplasmic immunostaining were separately evaluated on the entire cell-line population at 200× according to a semiquantitative score system (Histoscore). Percentages of positive EGFR cells were scored according to these cut-off values: < 1% = 0, > 1% < 25% = 1, > 25% < 50% = 2, > 50% < 75% = 3, > 75% = 4. Staining intensity was graded as 0 (negative), l (weak), 2 (moderate), 3 (strong). The percentage and staining intensity mean value product (0–12) gave us the final score classified as follows: < 1 = Negative, ≥ 1 < 4 = Low; ≥ 4 < 8 = Intermediate, ≥ 8 High.

### Scanning electron microscopy (SEM)

HT-29 and Caco-2 cells were seeded into Lab-Tek four chamber permanox slides (Nunc, Naperville IL) at 4 × 10^4 ^cells per chamber and incubated for 5 days. The cells were then treated as with the cell viability assay. Two independent experiments and two replicates for untreated and treated cells were conducted per experiment.

SEM (Philips SEM 515, Eindhoven, The Netherlands) was performed to examine the cell morphology.

All the slides were delicately rinsed with PBS in order not to detach cells from the surfaces. Cells were fixed with Karnowsky solution (1.5 % glutaraldeyde, 1% paraformaldeyde, 0.1 M Cacodilate buffer) for 30 min, then the slides with adhering cells were rinsed three times with Cacodilate buffer 0.1 M, postfixed for 20 min with Os_2_O_4 _1% in Cacodilate buffer, dehydrated with ethanol and finally dried with 2× hexamethyldisilizane (HDMS) for 15 min.

The slides were mounted on stubs with carbon bi-adhesive film, covered with a 20 nm-thick gold-palladium film and observed at 15 kV.

### RNA Extraction, Hybridization on cDNA Arrays, DNA microarray screening and analysis

The experimental procedures and data are available at  according to the Minimum Information About a Microarray Experiment standards [accession code no. GSE8967].

### Array image and data analysis

A GenePix 4000a DNA microarray scanner (Axon, Union City, CA, USA) was used to scan the slides under dried conditions. The laser power for scanning green and red colours was adjusted in order to obtain a global intensity ratio near to 1. If necessary, further washes were performed to reduce the non-specific background.

Each spot was defined using the grid schema provided by the manufacturer, with manual adjustment for the positioning of spot blocks. Spots showing no signal or obvious defects were accordingly flagged by visual inspection and excluded from analysis.

All statistical analyses on microarray data were performed using R software v2.5.0  and the Bioconductor software package . The microarray data were initially background-corrected using a normal plus exponential convolution model, normalized a) within arrays using a method that normalizes the M-values for each single microarray using robustly fitted regression splines for each print-tip group and an empirical Bayesian approach in order to shrink the individual print-tip curves towards a common value, and subsequently b) between arrays using a method which ensures that the A-values (average intensities) have the same empirical distribution across arrays, leaving the M-values (log-ratios) unchanged [39].

After the normalization step the probes were pre-filtered on the basis of empty spots and negative control intensity distribution over all the arrays. A threshold of log intensity = 6.2 was chosen. On this basis 16,443 out of 20,160 probes showing a mean intensity > 6.2 in at least one sample were considered for further analysis.

Hierarchical agglomerative clustering was performed on the correlation distance between samples.

Separate channel analysis was applied to the dataset; a mixed linear model was fitted to data after estimating the correlation between the two channels for the same spot.

A moderated t statistic was computed using an empirical Bayes method to shrink the probe-wise sample variances towards a common value and to augment the degrees of freedom for individual variances [40]. The Benjamini Hochberg method for multiple tests was used to obtain an adjusted p value.

Pathway analysis: affected biological pathways were defined according to the KEGG annotation [41] and mapping between probes and pathways was accomplished by querying the KEGG Database via R software. For each pathway *P *significance analysis was calculated considering the hypergeometric distribution [42]:

(1) $$p=\frac{S!F!N_{P}!N_{\bar{P}}!}{\alpha !\beta !\gamma !\delta !N!}$$

where

*α *= number of significant probes ∈ *P.*

*β *= number of non significant probes∈ *P*.

*γ *= number of significant probes ∉ *P*.

*δ *= number of non significant probes ∉ *P*.

*S *= number of significant probes in the array.

*F *= number of non significant probes in the array.

*N*_*P *_= number of probes ∈ *P*.

$N_{\bar{P}}$ = number of probes ∉ *P*.

The pathway P was considered significant if p ≤ 0.05.

We performed a pathway analysis which leads to more robust, reproducible results and easier biological interpretation. At the same time it represents an alternative way of post hoc analysis, relaxing the significant threshold for single genes without applying any severe statistical correction for multiple testing i.e. false discovery rate (FDR) [43,44]. By this approach we can take significant collective effects into consideration even if each gene in the group is not particularly significant from a statistical point of view [45,46].

Finally we defined heat maps as: graphical representations of selected microarray data showing the expression level of selected genes across a number of comparable cells under different treatments.

## Results

We characterized HT-29 and Caco-2 cell lines according to their viability, cell cycle, EGFR expression and cell morphology in untreated and treated conditions in order to compare their behavior and correlate their gene expression profiles changes with experimental conditions.

### Cell-viability assay

**Figure 1:**
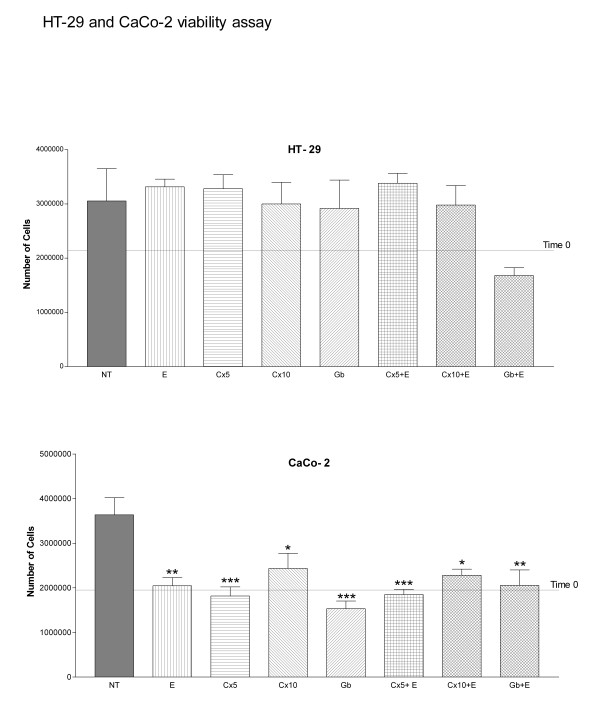
**HT-29 and Caco-2 viability assay**. Untreated (NT); 10 nM EGF (E); 5 (Cx5) and 10 (Cx10) nmol/L cetuximab; (Gb) 1 μmol/L gefitinib; 5 or 10 nmol/L cetuximab plus 10 nM EGF (Cx5 + E or Cx10 + E); 1 μmol/L gefitinib plus 10 nM EGF (Gb + E). ANOVA One-way analysis of variance and Tukey's Multiple Comparison Test. Caco-2. NT vs: **E, ***Cx5, *Cx10, ***Gb, ***Cx5 + E, *Cx10 + E, **Gb + E. *p < 0.05, **p < 0.01, ***p < 0.001. Each point represents a mean of quadruplicate values for each sample ± SD.

HT-29 was compared to Caco-2 regarding cell growth in normal conditions and after 24 hours of drug treatment. Caco-2 showed a statistically significant reduction in viability between controls and all treatments; no statistically significant differences were found in cell viability between untreated and treated HT-29 (Fig. 1).

### Cell-cycle analysis

Flow cytometry analysis was performed to determine the influence of treatments on the HT-29 and Caco-2 cell cycle (Table 1). There were no statistically significant differences between treated and untreated cells for the G0/G1 phase (with the exception of untreated vs 10 nM EGF plus 10 nmol/L cetuximab, p < 0.05 in both cell lines and for Caco-2 vs EGF, p < 0.05). As regards the G2/M phase, it is remarkable that there are 2-fold differences for HT-29 and 3-fold differences for Caco-2 when cetuximab both at 5 nM and 10 nM plus EGF treatment is compared to gefitinib 1 μmol/L plus EGF. In particular it is interesting that for EGF treatment there are 2-fold differences between HT-29 (9.26 %) and Caco-2 (18.21%); besides these two values there are the following differences compared to the relative untreated cells: a 1.43-fold difference for HT-29 and a 0.75-fold difference for Caco-2.

**Table 1:** Cell cycle distribution (%)

**HT-29**	***G*_0_/*G*_1_**	***S***	***G*_2_/*M***
NT	59,24	27,52	13,24
E	53,82	36,92	9,26
Cx5	56,97	28,55	14,48
Cx10	65,01	27,06	7,93
Gb	55,08	32,88	12,04
Cx5+E	53,24*	30,08	16,68
Cx10+E	48,41	36,03	15,56
Gb+E	54,41	37,89	7,7

**Caco-2**	***G*_0_/*G***_1_	***S***	***G*_2_/*M***

NT	66,25	20	13,75
E	55,1*	26,69	18,21
Cx5	73,73	15,36	10,91
Cx10	72,50	15,38	12,12
Gb	70,04	18,5	11,46
Cx5+E	57,20	21,13	21,67
Cx10+E	56,01*	24,02	19,97
Gb+E	66,35	26,52	7,13

NT = untreated; E = Epidermal growth factor 10 nmol/L; Cx5 = cetuximab 5 nmol/L; Cx10 = cetuximab 10 nmol/L; Gb = gefitinib 1 μmol/L; Cx5 + E = cetuximab 5 nmol/L + Epidermal growth factor 10 nmol/L; Cx10 + E = cetuximab 10 nmol/L + Epidermal growth factor 10 nmol/L; Gb + E = gefitinib 1 μmol/L + Epidermal growth factor 10 nmol/L. **ANOVA One-way analysis of variance Tukey's Multiple Comparison Test. Each point represents a mean of quadruplicate values**.

*Statistically Significant

### Immunohistochemistry

**Figure 2:**
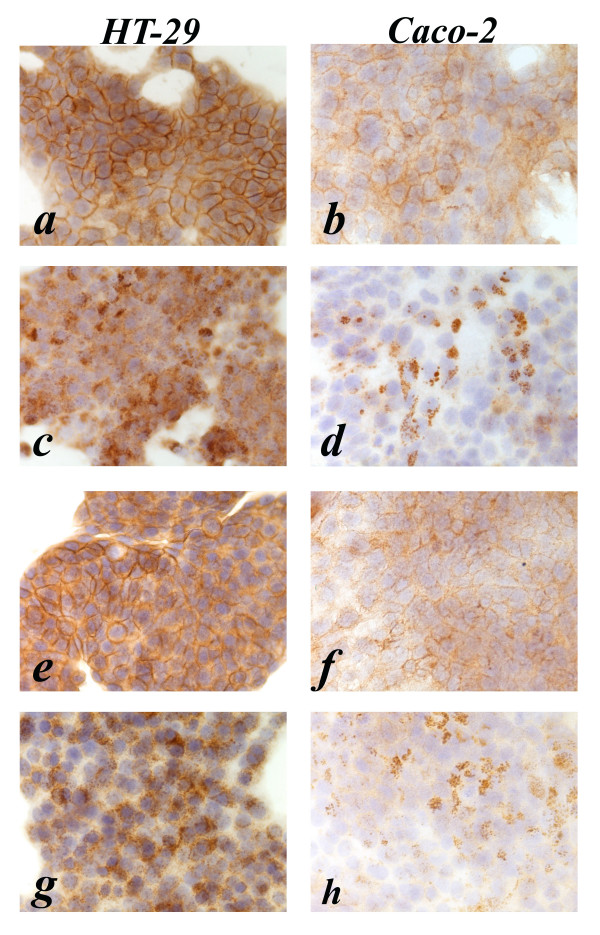
**Anti-EGFR immunostaining pattern in HT-29 and Caco-2 cell lines, respectively**. a, b (untreated); c, d (10 nM EGF treated); e, f (5 nmol/L cetuximab treated); g, h (5 nmol/L cetuximab plus 10 nM EGF treated).

The semiquantitative histoscore evaluation of the EGFR immunostaining is summarized in Table 2. Untreated cells showed a high EGFR expression for HT-29 and a moderate expression for Caco-2 (Fig. 2a, b).

**Table 2:** HT-29 and Caco-2 semiquantitative EGFR immunostaining evaluation (FS Cod)

	**HT-29**		**Caco-2**	
		
	Membrane	Cytoplasm	Membrane	Cytoplasm
NT	Intermediate	Intermediate	Low	Low
E	Negative	Intermediate	Negative	Low
Cx5	Intermediate	Intermediate	Low	Low
Cx5 + E	Negative	Intermediate	Negative	Low
Cx10	Intermediate	Low	Negative	Low
Cx10 + E	Negative	Intermediate	Negative	Low
Gb	Intermediate	Intermediate	Low	Low
Gb + E	Negative	Intermediate	Negative	Low

Membrane = Membrane immunostaining Histoscore; Cytoplasm = Cytoplasmic immunostaining Histoscore; FS Cod = Final Score coded according to cut-off values (see Material and Methods).

NT = untreated; E = Epidermal growth factor 10 nmol/L; Cx5 = cetuximab 5 nmol/L;

Cx10 = cetuximab 10 nmol/L; Gb = gefitinib 1 μmol/L; Cx5 + E = cetuximab 5 nmol/L + Epidermal growth factor 10 nmol/L; Cx10 + E = cetuximab 10 nmol/L + Epidermal growth factor 10 nmol/L;

Gb + E = gefitinib 1 μmol/L + Epidermal growth factor 10 nmol/L.

After treatment with EGF the EGFR immunostaining shows internalization of EGFR in both cell lines as revealed by the strong granular cytoplasmic immunostaining in HT-29 and, to a lesser degree, in Caco-2 cells, without any observable membrane staining (Fig. 2c, d). After treatment with 5 nM cetuximab the EGFR immunostaining shows continuous moderate to strong membrane staining of HT-29 cells and continuous but weak membranous brown staining of Caco-2 cells (Fig. 2e, f).

**Figure 3:**
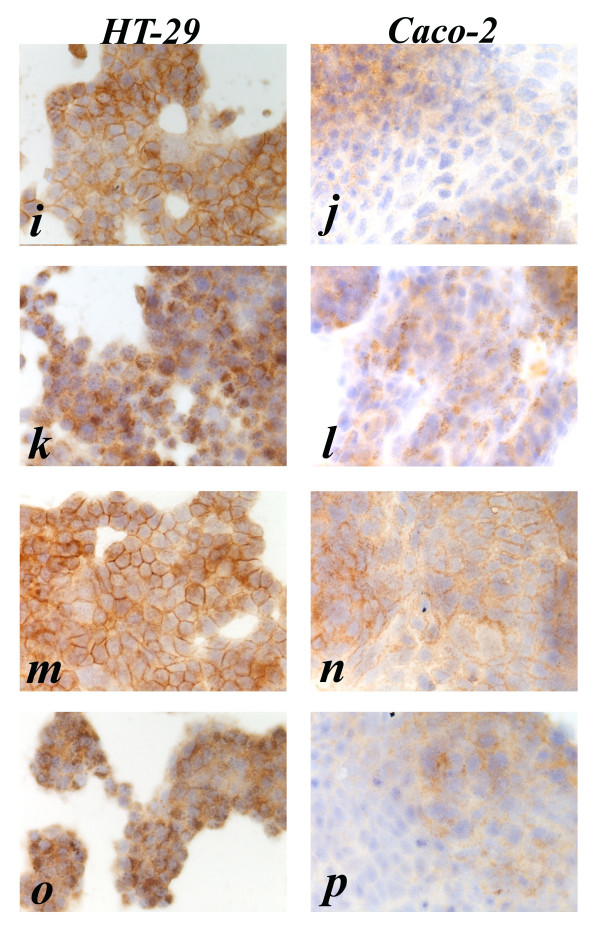
**Anti-EGFR immunostaining pattern in HT-29 and Caco-2 cell lines, respectively**. i, j (10 nmol/L cetuximab treated); k, l (10 nmol/L cetuximab plus 10 nM EGF treated); m, n (1 μmol/L gefitinib treated); o, p (1 μmol/L gefitinib plus 10 nM EGF treated). HT-29. Continuous moderate to strong membrane staining is present in untreated and cetuximab or gefitinib treated cells. Strong granular cytoplasmic immunostaining was present for all treatments plus EGF, without any observable membrane staining. Caco-2. Continuous weak to moderate membrane brown staining is present in untreated as well as in cetuximab 5 nmol/L and gefitinib 1 μmol/L treated cells. Weak membrane immunostaining was present in cetuximab 10 nmol/L treated cells. The cytoplasmic immunostaining pattern was granular in EGF 10 nM, diffuse in gefitinib 1 μmol/L plus EGF 10 nM and a mixture of the two (granular and diffuse) in cetuximab 5 and 10 nmol/L plus EGF 10 nM treated cells.

After combination treatment with 5 nM cetuximab plus EGF the EGFR immunostaining shows strong and diffuse granular cytoplasmic immunostaining in HT-29 cells, and weak and focal cytoplasmic granular staining in Caco-2 cells (Fig. 2g, h). After treatment with 10 nM cetuximab the EGFR immunostaining shows diffuse moderate membranous staining of HT-29 cells and weak membranous decoration of Caco-2 cells (Fig. 3i, j). After combination treatment with 10 nM cetuximab plus EGF the EGFR immunostaining shows strong granular cytoplasmic staining in HT-29 cells and weak cytoplasmic granular staining of Caco-2 cells (Fig. 3k, l). After treatment with gefitinib the EGFR immunostaining shows diffuse and strong membranous staining of HT-29 cells, and focal weak immunostaining of Caco-2 cells (Fig. 3m, n). Finally after combination treatment with gefitinib plus EGF the EGFR immunostaining shows strong granular cytoplasmic immunostaining of HT-29 and focal and weak cytoplasmic staining of Caco-2 (Fig. 3o, p).

In summary, Caco-2 cells displayed reduced immunostaining for EGFR when compared to HT-29 cells, while both cell types became negative for membrane staining following treatment with EGF, cetuximab plus EGF or gefitinib plus EGF.

### Scanning electron microscopy (SEM)

**Figure 4:**
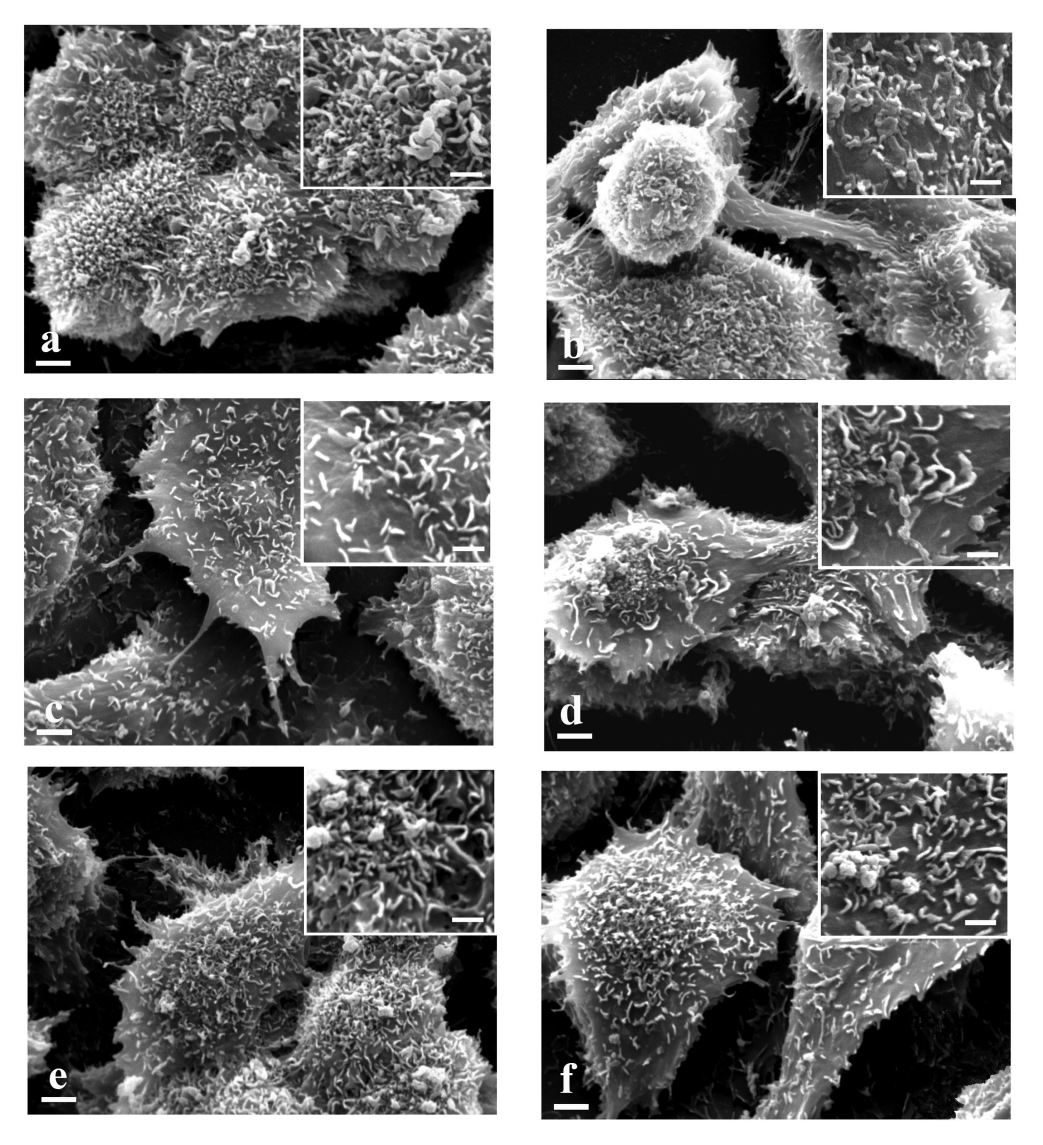
**SEM ×4000 (bar 2 μm)**. HT-29 and inserts (×8000) (bar 1 μm). a. untreated cells. Evident cellular boundaries. Insert: short microvilli are present. b. EGF treated cells. Same morphology as untreated cells. c. 10 nmol/L cetuximab treated cells. Filopodi are evident. Insert: microvilli reduction is evident. d. 10 nmol/L cetuximab plus 10 nM EGF treated cells. Filipodi and some vesicles are evident. Insert: microvilli reduction is evident. e. 1 μmol/L gefitinib treated cells. Some vesicles are evident. Insert: microvilli reduction is evident. f. 1 μmol/L gefitinib plus 10 nM EGF treated cells. Lamellipodi, some vesicles and weak contacts with nearby cells are evident. Insert: microvilli reduction is evident.

**Figure 5:**
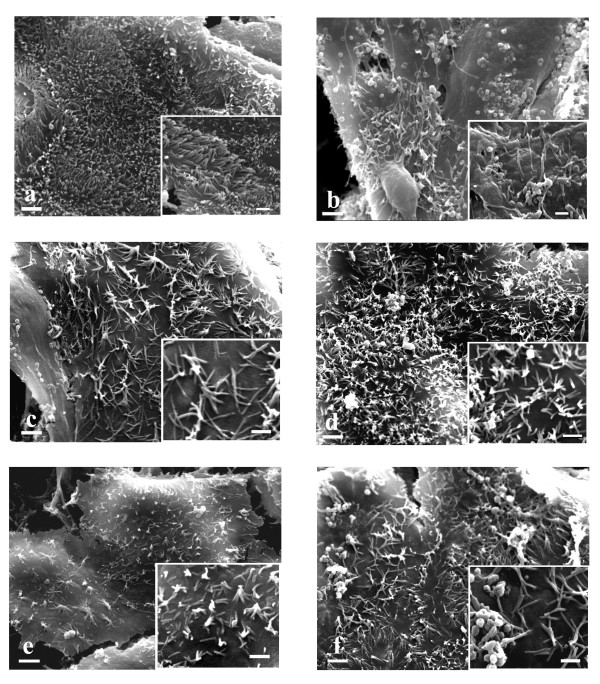
**SEM ×4000 (bar 2 μm)**. Caco-2 and inserts (×8000) (bar 1 μm). a. untreated cells. A large number of microvilli are evident. The cellular boundaries do not appear. Insert: long microvilli are present. b. EGF treated cells. A lot of vesicles are evident. Insert: a small number of microvilli are present. c. 10 nmol/L cetuximab treated cells. The microvilli diminish in number and lose their erect position. Insert: microvilli make contacts forming a star morphology. d. 10 nmol/L cetuximab plus 10 nM EGF treated cells. Same morphology as 10 nmol/L cetuximab treated cells. e. 1 μmol/L gefitinib treated cells. The microvilli diminish in number and lose their erect position. Insert: microvilli make contacts forming a star morphology. f. 1 μmol/L gefitinib plus 10 nM EGF treated cells. Same morphology as 1 μmol/L gefitinib treated cells.

HT-29 showed a different morphology from Caco-2. They were generally smaller than Caco-2, their cellular boundaries appeared more evident (Fig. 4a and 5a) and their microvilli were shorter than those of Caco-2 (Fig. 4a and 5a inserts).

HT-29 cells treated with EGF did not show any morphological differences from untreated cells, while Caco-2 displayed a lot of vesicles and microvilli reduction. Cellular boundaries were more evident (Fig. 4b and 5b and inserts). In Caco-2 EGFR binds EGF and the evidence of this binding could be microvilli transformation in a lot of vesicles.

HT-29 cells treated with 5 nM cetuximab lose their contacts with other cells and show filipodi and microvilli reduction. The Caco-2 counterparts showed microvilli reduction. They lost their erect position and made contacts with each other on their apical surface forming a star morphology.

HT-29 and Caco-2 cells treated with 10 nM cetuximab showed the same morphology as when treated with 5 nM cetuximab (Fig. 4c and 5c and inserts). Both HT-29 and Caco-2 displayed morphological transformations with cetuximab treatment. The high microvilli reduction in both cellular lines is an indication of EGFR-cetuximab binding. The loss of HT-29 cellular contacts and the presence of filipodi are clear signals of differentiation toward apoptosis. In Caco-2 the microvilli orientation changed to form star clusters.

HT-29 cells treated with 5 nM cetuximab plus EGF lost their contacts with other cells and showed filipodi and microvilli reduction. Caco-2 here showed the same pattern as cetuximab alone. Some vesicles were present.

HT-29 cells treated with 10 nM cetuximab plus EGF showed some vesicles and the same morphology as seen when treated with 5 nM cetuximab plus EGF. This finding is also observed with Caco-2 cells (Fig. 4d and 5d and inserts). The morphological transformations of both cellular lines after this treatment showed an accumulation of the effects of EGF and cetuximab used separately.

HT-29 cells treated with gefitinib displayed some vesicles and a reduced number of microvilli. Caco-2 likewise showed some vesicles and pronounced microvilli reduction. The microvilli lost their erect position and made contacts with each other on their apical surface forming a star morphology (Fig. 4e and 5e and inserts). Gefitinib induced a morphological transformation in both HT-29 and Caco-2. In particular Caco-2 showed the same morphology as induced by cetuximab treatment, but more pronounced; on the contrary morphological modifications to HT-29 were less evident than for cetuximab treatment.

HT-29 cells treated with gefitinib plus EGF proved to have plasmatic membranes with lamellipodi and weak contacts with nearby cells. Some vesicles were present. The Caco-2 counterparts showed the same behavior as with cetuximab treatment (Fig. 4f and 5f and inserts). The morphological transformations of the 2 cellular lines after this treatment presented a cumulative picture of the effects of EGF and gefitinib used separately.

### DNA microarray data analysis

The two cell lines responded to the different types of treatment with changes in gene expression profiling affecting a large number of genes that showed a fold change greater than 2-fold (Table 3). For HT-29 gefitinib treatment affected a greater number of genes (885 up-regulated and 1253 down-regulated) than with Caco-2 where it was EGF treatment that affected the greater number of genes (915 up-regulated and 1134 down-regulated). The number of at least 2-fold up-regulated genes in both Caco-2 and HT-29 was: 124 for EGF treatment, 49 for cetuximab treatment, 138 for gefitinib treatment, 10 for gefitinib plus EGF treatment and none for cetuximab plus EGF treatment. The at least 2-fold down-regulated genes were: 274 for EGF treatment, 58 for cetuximab, 113 for gefitinib, 3 for cetuximab plus EGF and 32 for gefitinib plus EGF. Interestingly, for treatments plus EGF there were fewer genes affected than occurred in single treatments. A competition effect is possible, particularly for Caco-2 and especially for cetuximab plus EGF treatment.

**Table 3:** Number of genes altered as a function of the treatment type

Treatment type	E		Cx10		Gb		Cx10 + E		Gb + E	
					
	Down	Up	Down	Up	Down	Up	Down	Up	Down	Up
**HT-29**	855	544	465	287	1253	885	217	459	799	582
**Caco-2**	1134	915	541	731	577	868	238	57	226	64
Intersection	274	124	58	49	113	138	3	0	32	10

E = Epidermal growth factor 10 nmol/L; Cx10 = cetuximab 10 nmol/L; Gb = gefitinib 1 μmol/L;

Cx10 + E = cetuximab 10 nmol/L + Epidermal growth factor 10 nmol/L; Gb + E = gefitinib 1 μmol/L + Epidermal growth factor 10 nmol/L; Down = 1/2 fold; Up = 2 fold.

For all treatments we also identified the significantly involved pathways in HT-29 and Caco-2 using the hypergeometric test described above (Table 4). Remarkably, for HT-29 we found the following pathways with EGF treatment: ubiquitine-mediated proteolysis and mTOR signaling, strictly related to MAPK signaling. For cetuximab treatment an interesting down-regulated pathway was calcium signaling related respectively to MAPK signaling, apoptosis and the phosphatidylinositol signaling system. We found that genes like *ITPR3 *(Inositol 1,4,5-triphosphate receptor) and *PLCD *(Phospholipase C, delta 4) were down-regulated. For gefitinib treatment we identified mTOR, the MAPK signaling, tight junction, cell communication and adherent junction pathways which are always down-regulated by this treatment.

**Table 4:** Pathways significantly represented in the single lines

Treatment type	p-val	Pathway affected
**HT-29**		
E	0,0127	Parkinson's disease
	0,0216	Bisphenol A degradation
	0,027	Nucleotide sugars metabolism
	0,028	ECM-receptor interaction
	0,029	Ubiquitin mediated proteolysis
	0,03	Neurodegenerative disorders
	0,03	Prion disease
	0,03	mTOR signaling pathway
		
Cx10	0,0001	Ribosome
	0,0288	Calcium signaling pathway
	0,0467	Prion disease
		
Gb	0,0056	T cell receptor signaling pathway
	0,0115	mTOR signaling pathway
	0,0163	Natural killer cell mediated cytotoxicity
	0,0187	Pentose and glucuronate interconversions
	0,0202	Tight junction
	0,0214	Starch and sucrose metabolism
	0,0242	Insulin signaling pathway
	0,0293	Long-term potentiation
	0,0332	GnRH signaling pathway
	0,0352	TGF-beta signaling pathway
	0,0402	MAPK signaling pathway
	0,0482	D-Glutamine and D-glutamate metabolism
	0,0497	Cell Communication
		
Cx10 + E	0,0001	Oxidative phosphorylation
	0,0211	Benzoate degradation via hydroxylation
	0,0254	Chronic myeloid leukemia
	0,0277	Antigen processing and presentation
	0,0319	Ribosome
	0,0461	Notch signaling pathway
		
Gb + E	0,0002	Cholera – Infection
	0,0003	PPAR signaling pathway
	0,0075	Tyrosine metabolism
	0,0090	Fluorene degradation
	0,0126	Benzoate degradation via hydroxylation
	0,0197	Insulin signaling pathway
	0,0205	Fatty acid metabolism
	0,0208	Calcium signaling pathway
	0,0225	Neuroactive ligand-receptor interaction
	0,0338	Oxidative phosphorylation
	0,0351	Glycerolipid metabolism
	0,0385	Glioma
	0,0428	Urea cycle and metabolism of amino groups
	0,0442	Neurodegenerative disorders
	0,0442	Styrene degradation
	0,0442	Fatty acid biosynthesis
	0,0445	1- and 2-Methylnaphthalene degradation
		
**Caco-2**		
E	0,0022	Epithelial cell signaling in Helicobacter pylori infection
	0,0034	Tight junction
	0,0133	Adherent junction
	0,0152	Dentatorubropallidoluysian atrophy (DRPLA)
	0,0179	Apoptosis
	0,0422	Methionine metabolism
	0,0436	D-Glutamine and D-glutamate metabolism
	0,0445	Selenoamino acid metabolism
	0,0481	Glycan structures – biosynthesis 2
	0,0496	Toll-like receptor signaling pathway
		
Cx10	0,0008	Oxidative phosphorylation
	0,0134	Ribosome
	0,0179	Cell cycle
	0,0193	Metabolism of xenobiotics by cytochrome P450
	0,0356	Glycan structures – biosynthesis 1
	0,0489	O-Glycan biosynthesis
		
Gb	0,0001	Ribosome
	0,0106	Basal cell carcinoma
	0,0135	Cell Communication
	0,0308	Valine, leukine and isoleukine degradation
	0,0394	Fatty acid metabolism
		
Cx10 + E	0.0141	Gap junction
	0.0163	GnRH signaling pathway
	0.0169	ECM-receptor interaction
	0.0364	Vitamin B6 metabolism
		
Gb + E	0,0183	C5-Branched dibasic acid metabolism
	0,0369	Tight junction

E = Epidermal growth factor 10 nmol/L; Cx10 = cetuximab 10 nmol/L; Gb = gefitinib. 1 μmol/L;

Cx10 + E = cetuximab 10 nmol/L + Epidermal growth factor 10 nmol/L; Gb + E = gefitinib 1 μmol/L + Epidermal growth factor 10 nmol/L. The pathways were considered significant if P ≤ 0.05.

For gefitinib plus EGF we found the Cholera-infection relating to tight junction and calcium signaling pathways. In Caco-2 cells, for EGF treatment we found apoptosis, tight junction and epithelial cell signaling in Helicobacter pylori infection. For cetuximab treatment we detected the cell cycle pathway (related to MAPK signaling), and in particular we found some important genes down-regulated by this drug: cyclin A, cyclin H, p21 and p57 and histone deacetilase 2.

For gefitinib treatment, we identified basal cell carcinoma and cell communication pathways. For cetuximab plus EGF we found gap junction pathways and finally for gefitinib plus EGF we detected the tight junction pathway.

Interestingly, for HT-29 mTOR signaling was a pathway common to EGF and gefitinib treatments, while the calcium signaling pathway was detected in cetuximab and gefitinib plus EGF treatments. Gefitinib down-regulated the expression of some genes that are overexpressed in EGF treatment, like Ras suppressor protein 1, *RAB2A *(member RAS oncogene family), *TACSTD1 *(Tumor-associated calcium signal transducer 1), *MOAP1 *(Modulator of apoptosis 1), *CDC42BPB *(CDC42 binding protein kinase beta), *RAB5C *(member RAS oncogene family) and *RASL12 *(RAS-like family 12).

Finally, the tight junction pathway was common to HT-29 gefitinib treatment, as well as Caco-2 EGF and gefitinib plus EGF treatments. The cell communication pathway proved to be affected in both cell lines following gefitinib treatment, while the genes that we found activated by this treatment were above all cytoskeleton genes, like laminin, fibronectin, collagen and gap junction proteins.

Genes were only selected as differentially expressed if they were at least 2-fold up- or down-regulated in both cell lines following each treatment [see Additional file 1 at , accession code no. GSE8967].

### Global gene expression analysis by hierarchical agglomerative cluster maps

**Figure 6:**
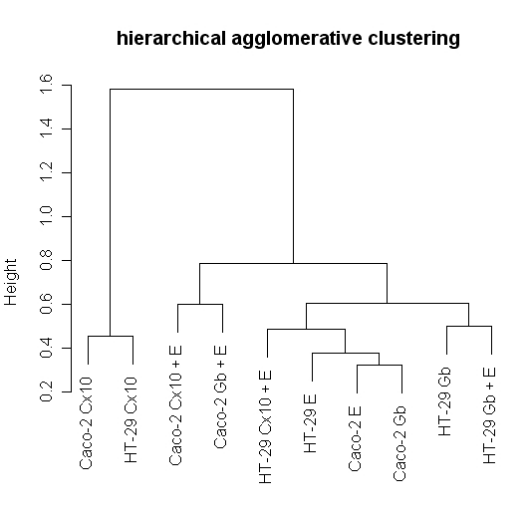
**Unsupervised agglomerative hierarchical clustering dendrograms**. Genes were selected for p-values < 0.05 adjusted for multiple testing by Benjamini and Hochberg's method. The clustering is based on the correlation distance between samples.

A comparison of treatment-induced changes in the global gene expression pattern was conducted on the two cell lines. In particular, the hierarchical agglomerative clustering procedure identified two main groups, one including Caco-2 and HT-29 treated with cetuximab, suggesting that the gene expression profile induced by cetuximab treatment is similar for the two lines. The other group was composed of all the rest divided into three subgroups. The most interesting subgroups were the one where Caco-2 treated with cetuximab in combination with EGF was associated with Caco-2 treated with gefitinib plus EGF and the subgroup where HT-29 treated with gefitinib was linked with HT-29 treated with gefitinib plus EGF. These associations may in the global view indicate a prevalence of cell-line specificity with respect to the treatment effects on gene expression profiles (Fig. 6).

### Cluster hierarchical Heat maps

For each treatment, we selected genes with p-values < 0.05 and a fold change above 2 or below 1/2 in both cell lines, while a comparative global heat map was constructed using an unsupervised hierarchical clustering method with a correlation distance between all the samples and between the genes selected. The EGF heat map shows that in both lines cetuximab treatment switched on genes that are switched off by EGF and, more weakly, by gefitinib, and vice versa. The cluster distribution deriving from these genes is interesting. Selection of the EGF gene target shows the matching of each type of treatment for the two cell lines, indicating that the kind of treatment accounted for more differences than the type of cell.

**Figure 7:**
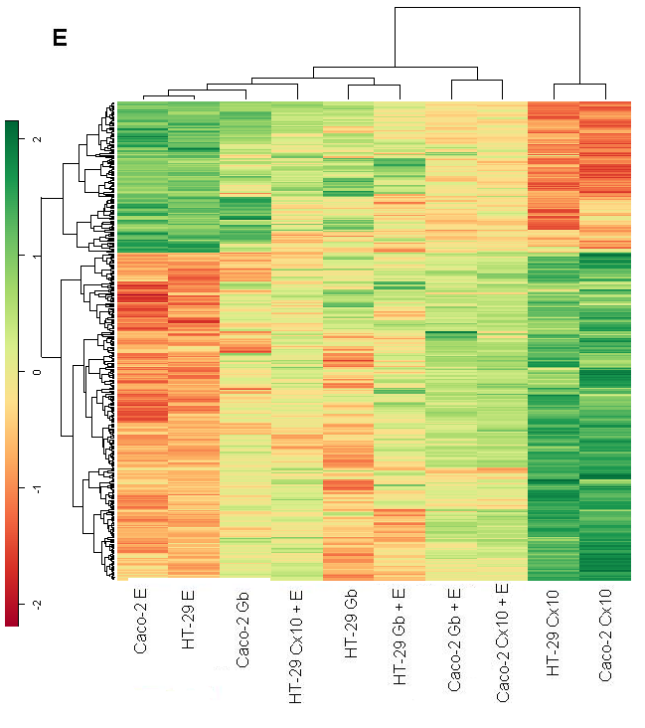
**Genes up-regulated with an expression ratio (comparing treated to untreated) greater than 2-fold and genes down-regulated with a ratio < 0.5 and p-values < 0.05, in HT-29 and Caco-2.** All heat maps were obtained by using an unsupervised hierarchical clustering method with a correlation distance between all the samples and between the selected genes. Heat map of genes selected from 10 nM EGF treated samples.

**Figure 8:**
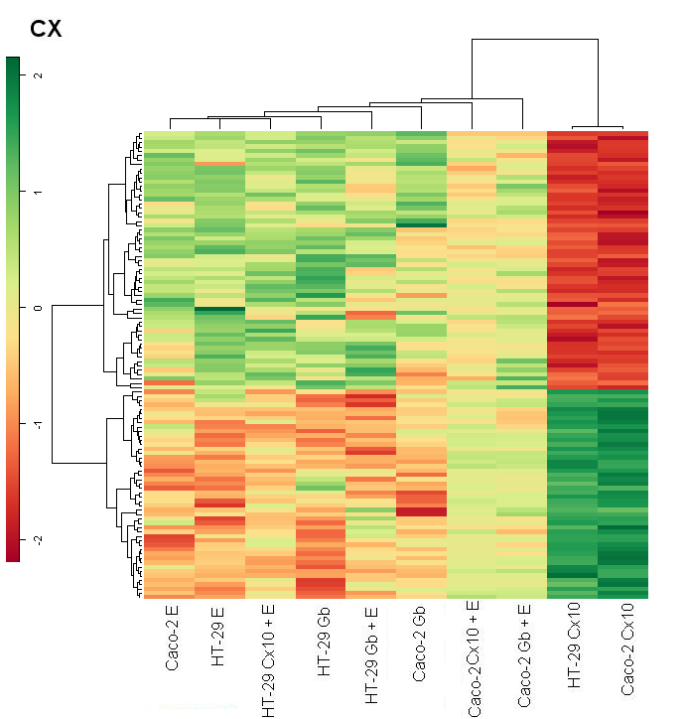
Heat map obtained from 10 nmol/L cetuximab treatment.

In the treatments with cetuximab or gefitinib plus EGF there is possible competition between one of the two drugs and EGF, because the genes appeared to be less stimulated than in the case of single drug treatment (Fig. 7). In the cetuximab heat map the Caco-2 line treated with cetuximab or gefitinib in association with EGF exhibits exactly the same pattern as in the EGF heat map but in a weaker fashion. The other group is mixed for treatment and cell line, indicating a weak stimulation, but with the opposite behavior in terms of gene on/off switching following cetuximab treatment (Fig. 8).

**Figure 9:**
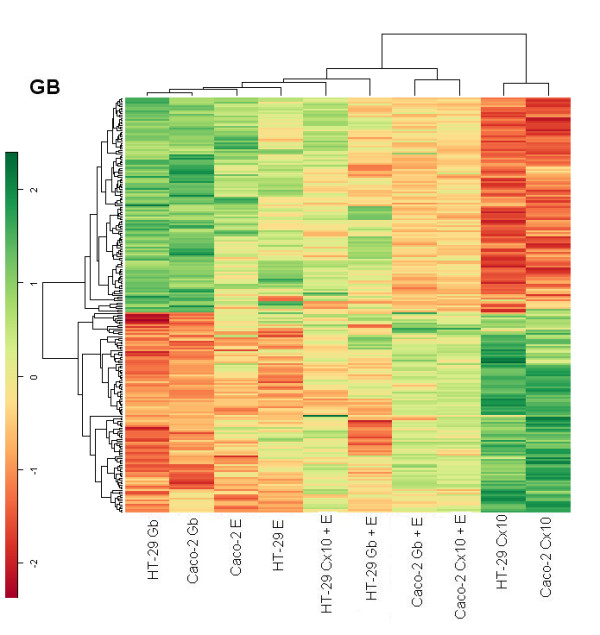
Heat map of the 1 μmol/L gefitinib treatment.

The gefitinib heat map shows that in the EGF heat map the same groups displayed a similar behavior, though it was more evident (Fig. 9).

Caco-2 and HT-29 cell lines treated with cetuximab showed a similar behavior in terms of on/off switched genes; while treatments with EGF or gefitinib showed the opposite pattern of expression for each heat map, indicating a different modulation of intensity induced for each cell line, depending on which gene was being considered in each heat map.

## Discussion

"EGFR is a pleiotropic signaler. The integrated biological response to EGFR activation varies from mitogenesis to apoptosis, migration, differentiation or de-differentiation even in the same cell depending on the context, which includes cell density, type of matrix, other cytokines, and even the position within a cell colony" [47]. ErbB ligands are numerous and classified into three major groups based on their direct binding to a particular erbB family member [3]. EGF, transforming growth factor-alpha and amphiregulin bind exclusively to erbB1[3].

In the present study we chose EGF among the natural ligands of EGFR. EGFR may also bind growth factors secreted by the epithelial cells themselves in an autocrine loop [48], which has been demonstrated for amphiregulin in the case of HT-29 [49] as well as Caco-2 [50] cells. The existence of autocrine loops was not considered in the present study and might be investigated in further works, but other authors [51] have used a fluorescence resonance energy transfer (FRET)-based method to measure the autonomous phosphorylation of HT-29 and Caco-2, and found that HT-29 showed FRET efficiencies over 50% and Caco-2 close to 30%.

To better study the possible responses after stimulation with EGF and the anti EGFR molecules cetuximab and gefitinib, we began by characterizing the two human colon cancer cell lines utilized (HT-29 and Caco-2) as working models.

First, we evaluated their level of EGFR expression about which the literature is broad-ranging but conflicting [51-55]. By immunohistochemistry we observed that HT-29 presents a higher level of EGFR expression than Caco-2, which proves weak to moderate by comparison.

The G0/G1 cell-cycle phase for untreated cells is equal to or over 60 %, indicating a high general level of differentiated cells at the beginning of experiments.

As regards the morphology of HT-29 and Caco-2, we characterized them by scanning electron microscopy. The enterocytes are characterized by tight junctions between adjacent cells and by the brush border on the apical cell surface consisting of organized microvilli. Each microvillus contains a bundle of actin filaments associated with proteins like villin, fimbrin, etc., and is anchored to the subjacent filamentous terminal web. In colon cells the microtubules control vesicle-trafficking between the Golgi network and the plasma membrane. The cytoskeleton dynamically reorganizes the cell shape during cell life [56-64]. We here show important differences between these two lines. In particular, two important features lend themselves to evaluating the effects of drug treatment: 1) cellular adhesion between adjacent cells (more evident in Caco-2 than in HT-29); 2) the abundant presence of microvilli which are shorter in HT-29 than in Caco-2. The drug treatment concentrations recommended by the pharmaceutical industries were able to act on the phenotype within 24 hours, especially on the cellular plasma membrane and cytoskeleton arrangement. Generally, in Caco-2, where the EGFR is weak to moderately expressed, the efficacy of treatments is stronger. We may explain this behavior by the different levels of basal EGFR phosphorylation in the absence of exogenous growth factor, which was shown to be weaker in Caco-2 than in HT-29 cells [34]. For EGF treatment on Caco-2 an apoptotic effect might be suggested by the statistically significant cell reduction number and by sub-microscopic transformations (clear cellular boundaries and plasma membrane reshaping). This is also confirmed by the microarray data where the hypergeometric test displays apoptosis, tight junction and adherent junction pathways. For HT-29 we focused our attention particularly on ECM receptor interaction, mTor signaling and ubiquitine-mediated proteolysis pathways which might confirm EGFR internalization and degradation following this course (as shown by immunohistochemistry and from the vesicles displayed by SEM).

As regards the G2/M phase, the different EGF effect on the two cell lines could be inversely correlated to the different level of autophosphorylation of HT-29 and Caco-2 [34]. Interestingly in HT-29 cells immunohistochemistry membrane staining is negative, whereas cytoplasmic staining stays intermediate in all treatments with EGF (untreated cells have intermediate staining in both the cytoplasm and the membrane). It is difficult to explain this finding, which could suggest EGFR degradation of the cytoplasm along with or rather than internalization of it.

Following cetuximab treatment, HT-29 showed some apoptosis features (loss of boundary contacts and the presence of filipodi). HT-29 and Caco-2 show a sizable reduction in microvilli: this acquired feature is likely to invalidate any microvilli absorption function.

Cetuximab on Caco-2 affects the cell cycle pathway, as indicated by down-regulation of cyclin A, cyclin H, p21 and p57 and histone deacetilase 2, while in HT-29 the calcium signaling pathway, correlated with EGFR activation [65], proved down-regulated as confirmed by the genes ITPR3 (Inositol 1,4,5-triphosphate receptor) and PLCD (Phospholipase C, delta 4). Interestingly, in both cell lines, cetuximab treatment activates the expression of *TP53BP2*; this gene encodes a member of the *ASPP *(apoptosis-stimulating protein of p53) family of p53 interacting proteins, which is down-regulated in EGF treatment. Moreover many genes involved in oxidative phosphorylation such as many subunits of ATP syntase (*ATP5J2, ATP5L, ATP5E, ATP5G1, ATP5G2*) and many subunits of NADH dehydrogenase (*NDUFA10, NDUFB1, NDUFB4, NDUFB8, NDUFC2, NDUFS2, NDUFS7, NDUFV1*) are down-regulated, suggesting a prominent role by cetuximab in impairing mitochondrial function. Remarkably, the only two genes that are up-regulated are COX10 which is related to cytochrome c, and a lysosomial subunit of ATP syntase (*ATP6V1G2*). Mitochondria have a major role in apoptosis and cancer and there is some evidence that the impairment of respiratory function (but without a lack of cytochrome c release) is associated with increased sensitivity to apoptosis [66]. There is also evidence in the literature of the role of tyrosine kinase signaling in the regulation of mitochondrial oxidative phosphorylation [67].

The hierarchical agglomerative clustering procedure confirms that the gene expression profile induced by cetuximab treatment is similar for Caco-2 and HT-29. The EGF, gefitinib and cetuximab heat maps show cetuximab treatment switching genes on and off with an exactly inverse pattern to EGF treatment.

Monoclonal antibody cetuximab generally affected fewer pathways than gefitinib, in both cell lines. This too is consistent with the rationale behind this drug, which is a specific target of EGFR, whereas gefitinib is a non-specific tyrosine kinase inhibitor.

Gefitinib treatment showed the same immunohistochemical picture as untreated cells, which was to be expected from its molecular anti-EGFR strategy. In both cell lines SEM unexpectedly reveals a sizable reduction of the microvilli which in Caco-2 lose their erect position: probably these acquired features indicate a cellular defect in absorption function.

Remarkably gefitinib down-regulates the expression of some genes that are overexpressed in EGF treatment, like Ras suppressor protein 1, *RAB2A *(a member of the RAS oncogene family), *TACSTD1 *(Tumor-associated calcium signal transducer 1), *MOAP1 *(modulator of apoptosis 1), *CDC42BPB *(CDC42 binding protein kinase beta), *RAB5C *(a member of the RAS oncogene family) and *RASL12 *(RAS-like family 12) although the gefitinib heat map shows a lot of groups displayed in the EGF heat map with the same behavior.

For cetuximab and gefitinib plus EGF treatments it is remarkable that in the G2/M cell cycle phase there are 2-fold differences for HT-29 and 3-fold differences for Caco-2 when cetuximab plus EGF treatment is compared to gefitinib plus EGF. We could explain this behavior by some summation of single effect treatments, exactly the same as for the morphological transformations data of the 2 cellular lines after these treatments, in agreement with the microarray data analysis where the affected genes are fewer in number than the genes involved in single treatments. A competition effect is possible, particularly for Caco-2 and especially for cetuximab plus EGF treatment.

Interestingly, with EGF treatment we found that in Caco-2 cells one pathway affected is epithelial cell signaling in Helicobacter pylori infection, while with gefitinib plus EGF treatment the HT-29 Cholera-infection pathway is affected. These findings, along with the microvillous submicroscopic alterations evidenced by SEM with cetuximab or gefitinib treatments, indicate the possibility that the side effect of diarrhea, which may be present in patients treated with gefitinib, and to a far lesser extent in patients treated with cetuximab, could be related to microvillus alterations, although our experimental model is not representative of *in vivo *changes occurring in normal enterocytes exposed to these drugs and the point requires further investigation. This is the first study that has brought to light these cellular microvilli alterations and this result could be correlated with the finding that Enteropathogenic Escherichia coli (EPEC) induces a severe watery diarrhea through a process linked with the loss of absorptive microvilli [68].

In our experimental model it is difficult to correlate the global gene expression profile and tumor sensitivity or resistance to treatment with the EGFR inhibitors. Some authors have "found an inverse correlation between EGFR expression and activity and argue against post-translational regulation of EGFR expression. The observed inverse correlation of EGFR activity with EGFR expression suggests a negative feedback loop between EGFR activity and expression in colorectal cancer cell lines"[69]. In the case of heat map analysis, we selected only the genes affected by EGFR and found a strict correlation and specificity of gene expression responsiveness to the drugs, suggesting that this method is useful when analyzing the dynamics of gene profiles.

## Conclusion

EGF and EGFR inhibitor treatments generally cause an apoptotic effect on HT-29 and Caco-2.

Cell viability, cell cycle, SEM and microarray analysis data confirm the extreme differentiation process towards cellular death. Caco-2 proves more reactive to treatments than HT-29, maybe owing to the lesser degree of autophosphorylation. The gene expression profile of cetuximab treatment is similar for the two cell lines, unlike EGF and gefitinib. Microvillous submicroscopic transformations found after drug treatment could be considered important features for studying a possible absorption alteration of enterocytes. Finally for cetuximab and gefitinib plus EGF treatments it is interesting to have found a possible joint effect of single agents, suggested by cell cycle, SEM and microarray analysis data.

## Competing interests

The authors declare that they have no competing interests.

## Authors' contributions

RS, ML, PS with GU, GR, SZ, IM and MT designed the study. RS coordinated the study. RS, ML and GM performed cell line cultures, experimental treatments and microarray hybridizations. MV carried out cell-viability assays, cell-cycle analysis and interpreted the relative data, performing statistical analysis too. CC and DS performed the immunohistochemistry analysis, and interpreted and discussed the relative data. DM and AR performed the SEM analysis and, in collaboration with RS, interpreted the relative data. ML, MF, GC, FP and PS analyzed the microarray data, including statistical analysis, and interpreted the relative results. GU, GR, SZ, IM, MT, LG and DC collaborated over the "Discussion" section and critically reviewed the whole manuscript. DC participated in the discussion of immunohistochemistry data too. RS, ML, PS and DM drafted the whole manuscript. All authors read, discussed and approved the final manuscript.

## Pre-publication history

The pre-publication history for this paper can be accessed here:



## Supplementary Material

Additional file 1 Table 5. Click here for file
