# Supplementary material for: Displayed correlation between gene expression profiles and submicroscopic alterations in response to cetuximab, gefitinib and EGF in human colon cancer cell lines
Source: BMC Cancer. 2008 Aug 8;8:227. doi: 10.1186/1471-2407-8-227 (PMC2528013; doi:10.1186/1471-2407-8-227)
Supplement: Additional file 1 — Table 5. [file 1471-2407-8-227-S1.doc]

| Treatment  Table 5.  Significantly regulated genes | Gene | | Description | |
| --- | --- | --- | --- | --- |
| Down regulated Cx10 treatment | *USMG5* | | Upregulated during skeletal muscle growth 5 homolog (mouse) | |
|  | *URG4* | | Up-regulated gene 4 | |
|  | *ATP5L* | | ATP synthase, H+ transporting, mitochondrial F0 complex, subunit G | |
|  | *MGST3* | | Microsomal glutathione S-transferase 3 | |
|  | *SIN3B* | | SIN3 homolog B, transcription regulator (yeast) | |
|  | *GPR27* | | G protein-coupled receptor 27 | |
|  | *LOC152217* | | Hypothetical protein BC007882 | |
|  | *KIF1A* | | Kinesin family member 1A | |
|  | *C14orf2* | | Chromosome 14 open reading frame 2 | |
|  | *RPS23* | | Ribosomal protein S23 | |
|  | *KIAA0644* | | KIAA0644 gene product | |
|  | *C12orf34* | | Chromosome 12 open reading frame 34 | |
|  | *CASP4* | | Caspase 4, apoptosis-related cysteine peptidase | |
|  | *BCAS3* | | Breast carcinoma amplified sequence 3 | |
|  | *CYC1* | | Cytochrome c-1 | |
|  | *C19orf56* | | Chromosome 19 open reading frame 56 | |
|  | *MBP* | | Myelin basic protein | |
|  | *MGC16121* | | Hypothetical protein MGC16121 | |
|  | *ATP5G1* | | ATP synthase, H+ transporting, mitochondrial F0 complex, subunit C1 (subunit 9) | |
|  | *RPS6KB2* | | Ribosomal protein S6 kinase, 70kDa, polypeptide 2 | |
|  | *TMEM54* | | Transmembrane protein 54 | |
|  | *APBA3* | | Amyloid beta (A4) precursor protein-binding, family A, member 3 (X11-like 2) | |
|  | *C2orf24* | | Chromosome 2 open reading frame 24 | |
|  | *TMEM18* | | Transmembrane protein 18 | |
|  | *PEG10* | | Paternally expressed 10 | |
|  | *AP2S1* | | Adaptor-related protein complex 2, sigma 1 subunit | |
|  | *SERF2* | | Small EDRK-rich factor 2 | |
|  | *FBLN1* | | Fibulin 1 | |
|  | *HSH2D* | | Hematopoietic SH2 domain containing | |
|  | *ATP5E* | | ATP synthase, H+ transporting, mitochondrial F1 complex, epsilon subunit | |
|  | *FOXB1* | | Forkhead box B1 | |
|  | *HINT1* | | Histidine triad nucleotide binding protein 1 | |
|  | *LY6G6C* | | Lymphocyte antigen 6 complex, locus G6C | |
|  | *CHCHD6* | | Coiled-coil-helix-coiled-coil-helix domain containing 6 | |
| Up-regulated  Cx treatment | *NLK* | | Nemo-like kinase | |
|  | *NAT1* | | N-acetyltransferase 1 (arylamine N-acetyltransferase) | |
|  | *MGC13057* | | Hypothetical protein MGC13057 | |
|  | *PPP3CB* | | Protein phosphatase 3 (formerly 2B), catalytic subunit, beta isoform | |
|  | *SERPING1* | | Serpin peptidase inhibitor, clade G (C1 inhibitor), member 1, (angioedema, hereditary) | |
|  | *LGTN* | | Ligatin | |
|  | *NR4A2* | | Nuclear receptor subfamily 4, group A, member 2 | |
|  | *ACVR1* | | Activin A receptor, type I | |
|  | *ARMC5* | | Armadillo repeat containing 5 | |
|  | *TM9SF3* | | Transmembrane 9 superfamily member 3 | |
|  | *CDC42BPB* | | CDC42 binding protein kinase beta (DMPK-like) | |
|  | *CSDE1* | | Cold shock domain containing E1, RNA-binding | |
|  | *SRA1* | | Steroid receptor RNA activator 1 | |
|  | *GALR1* | | Galanin receptor 1 | |
|  | *CASC5* | | Cancer susceptibility candidate 5 | |
|  | *STK38* | | Serine/threonine kinase 38 | |
|  | *C11orf57* | | Chromosome 11 open reading frame 57 | |
|  | *MTA1* | | Metastasis associated 1 | |
|  | *SCAMP4* | | Secretory carrier membrane protein 4 | |
|  | *ZMAT3* | | Zinc finger, matrin type 3 | |
|  | *RORB* | | RAR-related orphan receptor B | |
|  | *WDR1* | | WD repeat domain 1 | |
|  | *FADS2* | | Fatty acid desaturase 2 | |
|  | *SGIP1* | | SH3-domain GRB2-like (endophilin) interacting protein 1 | |
|  | *MCM3APAS* | | MCM3 minichromosome maintenance deficient 3 (S. cerevisiae) associated protein antisense | |
|  | *TARBP2* | | Tar (HIV-1) RNA binding protein 2 | |
|  | *COG4* | | Component of oligomeric golgi complex 4 | |
|  | *RFX3* | | Regulatory factor X, 3 (influences HLA class II expression) | |
|  | *SHOX* | | Short stature homeobox | |
|  | *BACE1* | | Beta-site APP-cleaving enzyme 1 | |
|  | *HARS* | | Histidyl-tRNA synthetase | |
|  | *PAH* | | Phenylalanine hydroxylase | |
|  | *NIPBL* | | Nipped-B homolog (Drosophila) | |
|  | *OS9* | | Amplified in osteosarcoma | |
|  | *AHI1* | | Abelson helper integration site 1 | |
|  | *TP53BP2* | | Tumor protein p53 binding protein, 2 | |
| Down-regulated Gb treatment | *NACA* | | Nascent-polypeptide-associated complex alpha polypeptide | |
|  | *RPL7A* | | Ribosomal protein L7a | |
|  | *TACSTD1* | | Tumor-associated calcium signal transducer 1 | |
|  | *ARF4* | | ADP-ribosylation factor 4 | |
|  | *ULBP3* | | UL16 binding protein 3 | |
|  | *RAB5C* | | RAB5C, member RAS oncogene family | |
|  | *ILF2* | | Interleukin enhancer binding factor 2, 45kDa | |
|  | *RGS6* | | Regulator of G-protein signalling 6 | |
|  | *TARBP2* | | Tar (HIV-1) RNA binding protein 2 | |
|  | *EIF4A2* | | Eukaryotic translation initiation factor 4A, isoform 2 | |
|  | *NOP5/NOP58* | | Nucleolar protein NOP5/NOP58 | |
|  | *INA* | | Internexin neuronal intermediate filament protein, alpha | |
|  | *YWHAE* | | Tyrosine 3-monooxygenase/tryptophan 5-monooxygenase activation protein, epsilon polypeptide | |
|  | *GALR1* | | Galanin receptor 1 | |
|  | *ISL1* | | ISL1 transcription factor, LIM/homeodomain, (islet-1) | |
|  | *EIF4A1* | | Eukaryotic translation initiation factor 4A, isoform 1 | |
|  | *ZMAT2* | | Zinc finger, matrin type 2 | |
|  | *HNRPC* | | Heterogeneous nuclear ribonucleoprotein C (C1/C2) | |
|  | *GNAS* | | GNAS complex locus | |
|  | *CDC42BPB* | | CDC42 binding protein kinase beta (DMPK-like) | |
|  | *RSU1* | | Ras suppressor protein 1 | |
|  | *C2orf25* | | Chromosome 2 open reading frame 25 | |
|  | *RASL12* | | RAS-like, family 12 | |
|  | *EIF4B* | | Eukaryotic translation initiation factor 4B | |
|  | *CAPN10* | | Calpain 10 | |
|  | *TSKS* | | Testis-specific kinase substrate | |
|  | *ATP6V1G2* | | ATPase, H+ transporting, lysosomal 13kDa, V1 subunit G2 | |
|  | *YWHAZ* | | Tyrosine 3-monooxygenase/tryptophan 5-monooxygenase activation protein, zeta polypeptide | |
|  | *TTC1* | | Tetratricopeptide repeat domain 1 | |
|  | *APOA5* | | Apolipoprotein A-V | |
|  | *DPM1* | | Dolichyl-phosphate mannosyltransferase polypeptide 1, catalytic subunit | |
|  | *PCBD2* | | Pterin-4 alpha-carbinolamine dehydratase/dimerization cofactor of hepatocyte nuclear factor 1 alpha (TCF1) 2 | |
|  | *CDKN3* | | Cyclin-dependent kinase inhibitor 3 (CDK2-associated dual specificity phosphatase) | |
|  | *ADSS* | | Adenylosuccinate synthase | |
|  | *ATP5A1* | | ATP synthase, H+ transporting, mitochondrial F1 complex, alpha subunit 1, cardiac muscle | |
|  | *SCFD1* | | Sec1 family domain containing 1 | |
|  | *VPS4B* | | Vacuolar protein sorting 4 homolog B (S. cerevisiae) | |
|  | *NFE2L2* | | Nuclear factor (erythroid-derived 2)-like 2 | |
|  | *MMAB* | | Methylmalonic aciduria (cobalamin deficiency) cblB type | |
|  | *BPTF* | | Bromodomain PHD finger transcription factor | |
|  | *HARS* | | Histidyl-tRNA synthetase | |
|  | *OTUD5* | | OTU domain containing 5 | |
|  | *C9orf7* | | Chromosome 9 open reading frame 7 | |
|  | *KIAA0737* | | KIAA0737 | |
|  | *GNPAT* | | Glyceronephosphate O-acyltransferase | |
|  | *MGAT2* | | Mannosyl (alpha-1,6-)-glycoprotein beta-1,2-N-acetylglucosaminyltransferase | |
|  | *CSDE1* | | Cold shock domain containing E1, RNA-binding | |
|  | *POGK* | | Pogo transposable element with KRAB domain | |
|  | *MCM4* | | MCM4 minichromosome maintenance deficient 4 (S. cerevisiae) | |
|  | *CASP4* | | Caspase 4, apoptosis-related cysteine peptidase | |
|  | *KRT10* | | Keratin 10 (epidermolytic hyperkeratosis; keratosis palmaris et plantaris) | |
|  | *ITPR3* | | Inositol 1,4,5-triphosphate receptor, type 3 | |
|  | *TCOF1* | | Treacher Collins-Franceschetti syndrome 1 | |
|  | *UGDH* | | UDP-glucose dehydrogenase | |
|  | *NFATC2IP* | | Nuclear factor of activated T-cells, cytoplasmic, calcineurin-dependent 2 interacting protein | |
|  | *PSMB10* | | Proteasome (prosome, macropain) subunit, beta type, 10 | |
|  | *RAB2A* | | RAB2A, member RAS oncogene family | |
|  | *PTPN22* | | Protein tyrosine phosphatase, non-receptor type 22 (lymphoid) | |
|  | *LAMB3* | | Laminin, beta 3 | |
|  | *UBE2E3* | | Ubiquitin-conjugating enzyme E2E 3 (UBC4/5 homolog, yeast) | |
|  | *CDC2L2* | | Cell division cycle 2-like 2 (PITSLRE proteins) | |
|  | *UGCGL1* | | UDP-glucose ceramide glucosyltransferase-like 1 | |
|  | *SERPINA1* | | Serpin peptidase inhibitor, clade A (alpha-1 antiproteinase, antitrypsin), member 1 | |
|  | *LAIR2* | | Leukocyte-associated immunoglobulin-like receptor 2 | |
|  | *BTG1* | | B-cell translocation gene 1, anti-proliferative | |
|  | *MOAP1* | | Modulator of apoptosis 1 | |
|  | *SLC15A2* | | Solute carrier family 15 (H+/peptide transporter), member 2 | |
|  | *HSDL1* | | Hydroxysteroid dehydrogenase like 1 | |
|  | *LGTN* | | Ligatin | |
|  | *PAX3* | | Paired box gene 3 (Waardenburg syndrome 1) | |
|  | *SGCG* | | Sarcoglycan, gamma (35kDa dystrophin-associated glycoprotein) | |
|  | *SLC25A31* | | Solute carrier family 25 (mitochondrial carrier; adenine nucleotide translocator), member 31 | |
|  | *PTPRC* | | Protein tyrosine phosphatase, receptor type, C | |
|  | *THRAP5* | | Thyroid hormone receptor associated protein 5 | |
|  | *TIAL1* | | TIA1 cytotoxic granule-associated RNA binding protein-like 1 | |
|  | *TOP2B* | | Topoisomerase (DNA) II beta 180kDa | |
|  | *MRPL3* | | Mitochondrial ribosomal protein L3 | |
|  | *GRHPR* | | Glyoxylate reductase/hydroxypyruvate reductase | |
|  | *PSMD13* | | Proteasome (prosome, macropain) 26S subunit, non-ATPase, 13 | |
|  | *SGIP1* | | SH3-domain GRB2-like (endophilin) interacting protein 1 | |
|  | *ADNP* | | Activity-dependent neuroprotector | |
|  | *STAT1* | | Signal transducer and activator of transcription 1, 91kDa | |
| Up-regulated  Gb treatment | *CNOT1* | | CCR4-NOT transcription complex, subunit 1 | |
|  | *ATP5L* | | ATP synthase, H+ transporting, mitochondrial F0 complex, subunit G | |
|  | *AMAC1L2* | | Acyl-malonyl condensing enzyme 1-like 2 | |
|  | *SLAMF1* | | Signaling lymphocytic activation molecule family member 1 | |
|  | *KCNG2* | | Potassium voltage-gated channel, subfamily G, member 2 | |
|  | *ALF* | | TFIIA-alpha/beta-like factor | |
|  | *LTBP4* | | Latent transforming growth factor beta binding protein 4 | |
|  | *PHLDB1* | | Pleckstrin homology-like domain, family B, member 1 | |
|  | *SLC32A1* | | Solute carrier family 32 (GABA vesicular transporter), member 1 | |
|  | *RNF135* | | Ring finger protein 135 | |
|  | *CRB1* | | Crumbs homolog 1 (Drosophila) | |
|  | *OTP* | | Orthopedia homolog (Drosophila) | |
|  | *ADAMTS14* | | ADAM metallopeptidase with thrombospondin type 1 motif, 14 | |
|  | *CDC42EP4* | | CDC42 effector protein (Rho GTPase binding) 4 | |
|  | *SIX1* | | Sine oculis homeobox homolog 1 (Drosophila) | |
|  | *TAOK2* | | TAO kinase 2 | |
|  | *FAM3A* | | Family with sequence similarity 3, member A | |
|  | *ZNF592* | | Zinc finger protein 592 | |
|  | *RPS23* | | Ribosomal protein S23 | |
|  | *PRSS27* | | Protease, serine 27 | |
|  | *APOBEC2* | | Apolipoprotein B mRNA editing enzyme, catalytic polypeptide-like 2 | |
|  | *KIF2C* | | Kinesin family member 2C | |
|  | *C12orf44* | | Chromosome 12 open reading frame 44 | |
|  | *HLA-E* | | Major histocompatibility complex, class I, E | |
|  | *TPCN1* | | Two pore segment channel 1 | |
|  | *ABCC6* | | ATP-binding cassette, sub-family C (CFTR/MRP), member 6 | |
|  | *SGK2* | | Serum/glucocorticoid regulated kinase 2 | |
|  | *TFE3* | | Transcription factor binding to IGHM enhancer 3 | |
|  | *SERF2* | | Small EDRK-rich factor 2 | |
|  | *SP2* | | Sp2 transcription factor | |
|  | *EPN3* | | Epsin 3 | |
|  | *ISG20L2* | | Interferon stimulated exonuclease gene 20kDa-like 2 | |
|  | *SCO1* | | SCO cytochrome oxidase deficient homolog 1 (yeast) | |
|  | *HSPC171* | | HSPC171 protein | |
|  | *BCAS3* | | Breast carcinoma amplified sequence 3 | |
|  | *FLYWCH1* | | FLYWCH-type zinc finger 1 | |
|  | *KCNH2* | | Potassium voltage-gated channel, subfamily H (eag-related), member 2 | |
|  | *TMEM161A* | | Transmembrane protein 161A | |
|  | *TBC1D13* | | TBC1 domain family, member 13 | |
|  | *RNASEH2A* | | Ribonuclease H2, subunit A | |
|  | *EDG8* | | Endothelial differentiation, sphingolipid G-protein-coupled receptor, 8 | |
|  | *ITFG2* | | Integrin alpha FG-GAP repeat containing 2 | |
|  | *TMEM35* | | Transmembrane protein 35 | |
|  | *CHD6* | | Chromodomain helicase DNA binding protein 6 | |
|  | *CRELD2* | | Cysteine-rich with EGF-like domains 2 | |
|  | *WNT10A* | | Wingless-type MMTV integration site family, member 10A | |
|  | *RASSF7* | | Ras association (RalGDS/AF-6) domain family 7 | |
|  | *ZNF160* | | Zinc finger protein 160 | |
|  | *POLR1E* | | Polymerase (RNA) I polypeptide E, 53kDa | |
|  | *CCRK* | | Cell cycle related kinase | |
|  | *IQSEC3* | | IQ motif and Sec7 domain 3 | |
|  | *ID1* | | Inhibitor of DNA binding 1, dominant negative helix-loop-helix protein | |
|  | *FLJ20433* | | Hypothetical protein FLJ20433 | |
|  | *MSI2* | | Musashi homolog 2 (Drosophila) | |
|  | *TSC22D2* | | TSC22 domain family, member 2 | |
|  | *PCMTD2* | | Protein-L-isoaspartate (D-aspartate) O-methyltransferase domain containing 2 | |
|  | *TCP10L* | | T-complex 10 (mouse)-like | |
|  | *ELK1* | | ELK1, member of ETS oncogene family | |
|  | *KLK11* | | Kallikrein-related peptidase 11 | |
|  | *PPP1R15A* | | Protein phosphatase 1, regulatory (inhibitor) subunit 15A | |
|  | *HLA-E* | | Major histocompatibility complex, class I, E | |
|  | *SNRPB* | | Small nuclear ribonucleoprotein polypeptides B and B1 | |
|  | *C5orf32* | | Chromosome 5 open reading frame 32 | |
|  | *SUSD4* | | Sushi domain containing 4 | |
|  | *ADM2* | | Adrenomedullin 2 | |
|  | *SPRY4* | | Sprouty homolog 4 (Drosophila) | |
|  | *SGCA* | | Sarcoglycan, alpha (50kDa dystrophin-associated glycoprotein) | |
|  | *ZNF206* | | Zinc finger protein 206 | |
|  | *C2orf24* | | Chromosome 2 open reading frame 24 | |
|  | *IGSF9B* | | Immunoglobulin superfamily, member 9B | |
|  | *ZNF703* | | Zinc finger protein 703 | |
|  | *SFRP1* | | Secreted frizzled-related protein 1 | |
|  | *LGP2* | | Likely ortholog of mouse D11lgp2 | |
|  | *OAZ3* | | Ornithine decarboxylase antizyme 3 | |
|  | *DUS3L* | | Dihydrouridine synthase 3-like (S. cerevisiae) | |
|  | *ARID1A* | | AT rich interactive domain 1A (SWI-like) | |
|  | *AP1GBP1* | | AP1 gamma subunit binding protein 1 | |
|  | *UBA52* | | Ubiquitin A-52 residue ribosomal protein fusion product 1 | |
|  | *TMEM54* | | Transmembrane protein 54 | |
|  | *SUPT3H* | | Suppressor of Ty 3 homolog (S. cerevisiae) | |
|  | *MRPL53* | | Mitochondrial ribosomal protein L53 | |
|  | *ZC3H12A* | | Zinc finger CCCH-type containing 12A | |
|  | *BMP10* | | Bone morphogenetic protein 10 | |
|  | *C12orf43* | | Chromosome 12 open reading frame 43 | |
|  | *SLC29A2* | | Solute carrier family 29 (nucleoside transporters), member 2 | |
|  | *INCENP* | | Inner centromere protein antigens 135/155kDa | |
|  | *LOC554223* | | Hypothetical LOC554223 | |
|  | *DKFZP434O047* | | DKFZP434O047 protein | |
|  | *FGF21* | | Fibroblast growth factor 21 | |
|  | *OTOF* | | Otoferlin | |
|  | *ARHGEF11* | | Rho guanine nucleotide exchange factor (GEF) 11 | |
|  | *FAM124B* | | Family with sequence similarity 124B | |
|  | *MARK4*  *SUV420H2* | | MAP/microtubule affinity-regulating kinase 4 | |
|  |  |  | Suppressor of variegation 4-20 homolog 2 (Drosophila) | |
| Down-regulated E treatment | | *TTC15* | | Tetratricopeptide repeat domain 15 |
|  | | *ATP6V1G2* | | ATPase, H+ transporting, lysosomal 13kDa, V1 subunit G |
|  | | *ZNF407* | | Zinc finger protein 407 |
|  | | *NUP43* | | Nucleoporin 43kDa |
|  | | *EXOC6B* | | Exocyst complex component 6B |
|  | | *PNN* | | Pinin, desmosome associated protein |
|  | | *TBX19* | | T-box 19 |
|  | | *INA* | | Internexin neuronal intermediate filament protein, alpha |
|  | | *PLA2G1B* | | Phospholipase A2, group IB (pancreas) |
|  | | *TRIM5* | | Tripartite motif-containing 5 |
|  | | *SLC25A31* | | Solute carrier family 25 (mitochondrial carrier; adenine nucleotide translocator), member 31 |
|  | | *NFATC2IP* | | Nuclear factor of activated T-cells, cytoplasmic, calcineurin-dependent 2 interacting protein |
|  | | *ARMC5* | | Armadillo repeat containing 5 |
|  | | *ABCC1* | | ATP-binding cassette, sub-family C (CFTR/MRP), member 1 |
|  | | *LAIR2* | | Leukocyte-associated immunoglobulin-like receptor 2 |
|  | | *LAMC3* | | Laminin, gamma 3 |
|  | | *PCNT* | | Pericentrin (kendrin) |
|  | | *ZMAT3* | | Zinc finger, matrin type 3 |
|  | | *RERE* | | Arginine-glutamic acid dipeptide (RE) repeats |
|  | | *RUNX2* | | Runt-related transcription factor 2 |
|  | | *ADAM30* | | ADAM metallopeptidase domain 30 |
|  | | *APOBEC1* | | Apolipoprotein B mRNA editing enzyme, catalytic polypeptide 1 |
|  | | *APOB* | | Apolipoprotein B (including Ag(x) antigen) |
|  | | *DYRK1A* | | Dual-specificity tyrosine-(Y)-phosphorylation regulated kinase 1A |
|  | | *POLR3G* | | Polymerase (RNA) III (DNA directed) polypeptide G (32kD) |
|  | | *DDEF1* | | Development and differentiation enhancing factor 1 |
|  | | *TACSTD1* | | Tumor-associated calcium signal transducer 1 |
|  | | *DNAJC1* | | DnaJ (Hsp40) homolog, subfamily C, member 1 |
|  | | *MYPN* | | Myopalladin |
|  | | *SPAG9* | | Sperm associated antigen 9 |
|  | | *C12orf11* | | Chromosome 12 open reading frame 11 |
|  | | *GPNMB* | | Glycoprotein (transmembrane) nmb |
|  | | *UBE2D1* | | Ubiquitin-conjugating enzyme E2D 1 (UBC4/5 homolog, yeast) |
|  | | *HAS2* | | Hyaluronan synthase 2 |
|  | | *MMAB* | | Methylmalonic aciduria (cobalamin deficiency) cblB type |
|  | | *DKFZp434N035* | | Hypothetical protein DKFZp434N035 |
|  | | *ENPEP* | | Glutamyl aminopeptidase (aminopeptidase A) |
|  | | *AGXT2L1* | | Alanine-glyoxylate aminotransferase 2-like 1 |
|  | | *ZNF432* | | Zinc finger protein 432 |
|  | | *SDC1* | | Syndecan 1 |
|  | | *CABLES2* | | Cdk5 and Abl enzyme substrate 2 |
|  | | *CDC42BPB* | | CDC42 binding protein kinase beta (DMPK-like) |
|  | | *MLN* | | Motilin |
|  | | *GALR1* | | Galanin receptor 1 |
|  | | *PSG9* | | Pregnancy specific beta-1-glycoprotein 9 |
|  | | *TXNIP* | | Thioredoxin interacting protein |
|  | | *PASK* | | PAS domain containing serine/threonine kinase |
|  | | *HGSNAT* | | Heparan-alpha-glucosaminide N-acetyltransferase |
|  | | *CTSL1* | | Cathepsin L |
|  | | *CRSP3* | | Cofactor required for Sp1 transcriptional activation, subunit 3, 130kD |
|  | | *GOLPH4* | | Golgi phosphoprotein 4 |
|  | | *ARG1* | | Arginase, liver |
|  | | *AHI1* | | Abelson helper integration site 1 |
|  | | *ENO1* | | Enolase 1, (alpha) |
|  | | *ARF4* | | ADP-ribosylation factor 4 |
|  | | *DYRK3* | | Dual-specificity tyrosine-(Y)-phosphorylation regulated kinase 3 |
|  | | *HIATL2* | | Hippocampus abundant gene transcript-like 2 |
|  | | *PLEKHF2* | | Pleckstrin homology domain containing, family F (with FYVE domain) member 2 |
|  | | *TARBP2* | | Tar (HIV-1) RNA binding protein 2 |
|  | | *SUMO2* | | SMT3 suppressor of mif two 3 homolog 2 (S. cerevisiae) |
|  | | *SAP30* | | Sin3A-associated protein, 30kDa |
|  | | *NR2E3* | | Nuclear receptor subfamily 2, group E, member 3 |
|  | | *SLC41A2* | | Solute carrier family 41, member 2 |
|  | | *PTPN2* | | Protein tyrosine phosphatase, non-receptor type 2 |
|  | | *SAMD4A* | | Sterile alpha motif domain containing 4° |
|  | | *RPL7A* | | Ribosomal protein L7a |
|  | | *EHD4* | | EH-domain containing 4 |
|  | | *PEPP-2* | | PEPP subfamily gene 2 |
|  | | *SKIP* | | Skeletal muscle and kidney enriched inositol phosphatase |
|  | | *RPP40* | | Ribonuclease P 40kDa subunit |
|  | | *CASP3* | | Caspase 3, apoptosis-related cysteine peptidase |
|  | | *ARHGEF6* | | Rac/Cdc42 guanine nucleotide exchange factor (GEF) 6 |
|  | | *MGC15705* | | Hypothetical protein MGC15705 |
|  | | *PCDHGC3* | | Protocadherin gamma subfamily C, |
|  | | *PVRL4* | | Poliovirus receptor-related 4 |
|  | | *PAIP1* | | Poly(A) binding protein interacting protein 1 |
|  | | *TCEA2* | | Transcription elongation factor A (SII), 2 |
|  | | *NRG1* | | Neuregulin 1 |
|  | | *MAP4K3* | | Mitogen-activated protein kinase kinase kinase kinase 3 |
|  | | *ARMC10* | | SVH protein |
|  | | *JAM3* | | Junctional adhesion molecule 3 |
|  | | *IFNA17* | | Interferon, alpha 17 |
|  | | *SUMO1* | | SMT3 suppressor of mif two 3 homolog 1 (S. cerevisiae) |
|  | | *DAB1* | | Disabled homolog 1 (Drosophila) |
|  | | *ISL1* | | ISL1 transcription factor, LIM/homeodomain, (islet-1 |
|  | | *TMED2* | | Transmembrane emp24 domain trafficking protein 2 |
|  | | *CHES1* | | Checkpoint suppressor 1 |
|  | | *NACA* | | Nascent-polypeptide-associated complex alpha polypeptide |
|  | | *OS9* | | Amplified in osteosarcoma |
|  | | *PGK2* | | Phosphoglycerate kinase 2 |
|  | | *MYO18B* | | Myosin XVIIIB |
|  | | *MCM4* | | MCM4 minichromosome maintenance deficient 4 (S. cerevisiae) |
|  | | *TSPAN13* | | Tetraspanin 13 |
|  | | *MAGEA10* | | Melanoma antigen family A, 1 |
|  | | *RTCD1* | | RNA terminal phosphate cyclase domain 1 |
|  | | *ILF2* | | Interleukin enhancer binding factor 2, 45kDa |
|  | | *KIAA1546* | | KIAA1546 |
|  | | *PPP2R2B* | | Protein phosphatase 2 (formerly 2A), regulatory subunit B, beta isofor |
|  | | *SGIP1* | | SH3-domain GRB2-like (endophilin) interacting protein 1 |
|  | | *SAC* | | Testicular soluble adenylyl cyclase |
|  | | *RGS6* | | Regulator of G-protein signalling 6 |
|  | | *SLC15A2* | | Solute carrier family 15 (H+/peptide transporter), member 2 |
|  | | *DCC* | | Deleted in colorectal carcinoma |
|  | | *RAB11FIP3* | | RAB11 family interacting protein 3 (class II) |
|  | | *IER2* | | Immediate early response 2 |
|  | | *ZNF638* | | Zinc finger protein 638 |
|  | | *CRAT* | | Carnitine acetyltransferase |
|  | | *PAX3* | | Paired box gene 3 (Waardenburg syndrome 1) |
|  | | *PEX1* | | Peroxisome biogenesis factor 1 |
|  | | *KIF26A* | | Kinesin family member 26A |
|  | | *RPS2* | | Ribosomal protein S2 |
|  | | *SPG3A* | | Spastic paraplegia 3A (autosomal dominant) |
|  | | *COL1A2* | | Collagen, type I, alpha 2 |
|  | | *PDHB* | | Pyruvate dehydrogenase (lipoamide) beta |
|  | | *FSCN3* | | Fascin homolog 3, actin-bundling protein, testicular (Strongylocentrotus purpuratus) |
|  | | *NUMB* | | Numb homolog (Drosophila) |
|  | | *PLS3* | | Plastin 3 (T isoform) |
|  | | *TCP11* | | T-complex 11 (mouse) |
|  | | *LRRFIP1* | | Leucine rich repeat (in FLII) interacting protein 1 |
|  | | *MATR3* | | Matrin 3 |
|  | | *PAH* | | Phenylalanine hydroxylase |
|  | | *ADH7* | | Alcohol dehydrogenase 7 (class IV), mu or sigma polypeptide |
|  | | *C14orf11* | | Chromosome 14 open reading frame 11 |
|  | | *ACTR3B* | | ARP3 actin-related protein 3 homolog B (yeast) |
|  | | *HPCAL4* | | Hippocalcin like 4 |
|  | | *ACVR1B* | | Activin A receptor, type IB |
|  | | *ITGB3BP* | | Integrin beta 3 binding protein (beta3-endonexin) |
|  | | *PDPK1* | | 3-phosphoinositide dependent protein kinase-1 |
|  | | *MATR3* | | Matrin 3 |
|  | | *CSDA* | | Cold shock domain protein A |
|  | | *LIFR* | | Leukemia inhibitory factor receptor alpha |
|  | | *ANXA5* | | Annexin A5 |
|  | | *TSPAN3* | | Tetraspanin 3 |
|  | | *CDS1* | | CDP-diacylglycerol synthase (phosphatidate cytidylyltransferase) 1 |
|  | | *BLK* | | B lymphoid tyrosine kinase |
|  | | *RAD54L2* | | RAD54-like 2 (S. cerevisiae) |
|  | | *METAP2* | | Methionyl aminopeptidase 2 |
|  | | *PRKAA2* | | Protein kinase, AMP-activated, alpha 2 catalytic subunit |
|  | | *DNAH9* | | Dynein, axonemal, heavy chain 9 |
|  | | *MGC10955* | | Hypothetical protein MGC10955 |
|  | | *UGT2B7* | | UDP glucuronosyltransferase 2 family, polypeptide B7 |
|  | | *TEF* | | Thyrotrophic embryonic factor |
|  | | *ZNF140* | | Zinc finger protein 140 |
|  | | *RARB* | | Retinoic acid receptor, beta |
|  | | *NPAS2* | | Neuronal PAS domain protein 2 |
|  | | *BHMT* | | Betaine-homocysteine methyltransferase |
|  | | *CRISPLD2* | | Cysteine-rich secretory protein LCCL domain containing 2 |
|  | | *REEP6* | | Receptor accessory protein 6 |
|  | | *TFCP2* | | Transcription factor CP2 |
|  | | *FLT3* | | Fms-related tyrosine kinase 3 |
|  | | *AHI1* | | Abelson helper integration site 1 |
|  | | *SCG2* | | Secretogranin II (chromogranin C) |
|  | | *PLD1* | | Phospholipase D1, phosphatidylcholine-specific |
|  | | *PCBD2* | | Pterin-4 alpha-carbinolamine dehydratase/dimerization cofactor of hepatocyte nuclear factor 1 alpha (TCF1) 2 |
|  | | *FGFR3* | | Fibroblast growth factor receptor 3 (achondroplasia, thanatophoric dwarfism |
|  | | *ASH1L* | | Ash1 (absent, small, or homeotic)-like (Drosophila |
|  | | *HIBCH* | | 3-hydroxyisobutyryl-Coenzyme A hydrolase |
|  | | *GNAS* | | GNAS complex locus |
|  | | *NR4A2* | | Nuclear receptor subfamily 4, group A, member 2 |
|  | | *YWHAZ* | | Tyrosine 3-monooxygenase/tryptophan 5-monooxygenase activation protein, zeta polipeptide |
|  | | *ING5* | | Inhibitor of growth family, member 5 |
|  | | *ZNF254* | | Zinc finger protein 254 |
|  | | *GRIN1* | | Glutamate receptor, ionotropic, N-methyl D-aspartate 1 |
|  | | *ANLN* | | Anillin, actin binding protein |
|  | | *MSR1* | | Macrophage scavenger receptor 1 |
|  | | *TIA1* | | TIA1 cytotoxic granule-associated RNA binding protein |
|  | | *YWHAQ* | | Tyrosine 3-monooxygenase/tryptophan 5-monooxygenase activation protein, theta polypeptide |
|  | | *BARX2* | | BarH-like homeobox 2 |
|  | | *EIF1AY* | | Eukaryotic translation initiation factor 1A, Y-linked |
|  | | *PLAG1* | | Pleiomorphic adenoma gene 1 |
|  | | *SNX4* | | Sorting nexin 4 |
|  | | *MGC4655* | | Hypothetical protein MGC4655 |
|  | | *CDC7* | | Cell division cycle 7 homolog (S. cerevisiae) |
|  | | *FUCA1* | | Fucosidase, alpha-L- 1, tissue |
|  | | *PDE4B* | | Phosphodiesterase 4B, cAMP-specific (phosphodiesterase E4 dunce homolog, Drosophila) |
|  | | *HARS* | | Histidyl-tRNA synthetase |
|  | | *ACPP* | | Acid phosphatase, prostate |
|  | | *GATA3* | | GATA binding protein 3 |
|  | | *HPS3* | | Hermansky-Pudlak syndrome 3 |
|  | | *TPST2* | | Tyrosylprotein sulfotransferase 2 |
|  | | *ARHGEF6* | | Rac/Cdc42 guanine nucleotide exchange factor (GEF) 6 |
|  | | *TP53BP2* | | Tumor protein p53 binding protein, 2 |
|  | | *GTF2I* | | General transcription factor II, i |
|  | | *ACTR8* | | ARP8 actin-related protein 8 homolog (yeast) |
|  | | *OSBPL1A* | | Oxysterol binding protein-like 1A |
|  | | *TNP1* | | Transition protein 1 (during histone to protamine replacement) |
|  | | *HIF1AN* | | Hypoxia-inducible factor 1, alpha subunit inhibitor |
|  | | *CCDC47* | | Coiled-coil domain containing 47 |
|  | | *RPS6KA5* | | Ribosomal protein S6 kinase, 90kDa, polypeptide 5 |
|  | | *SLC22A6* | | Solute carrier family 22 (organic anion transporter), member 6 |
|  | | *MYO5A* | | Myosin VA (heavy chain 12, myoxin) |
|  | | *VAT1* | | Vesicle amine transport protein 1 homolog (T. californica) |
|  | | *TCEA1* | | Transcription elongation factor A (SII), 1 |
|  | | *ACOX3* | | Coenzyme A oxidase 3, pristanoyl |
|  | | *LBX1* | | Ladybird homeobox homolog 1 (Drosophila) |
|  | | *C9orf77* | | Chromosome 9 open reading frame 77 |
|  | | *PIGQ* | | Phosphatidylinositol glycan anchor biosynthesis, class Q |
|  | | *CRKRS* | | Cdc2-related kinase, arginine/serine-rich |
|  | | *NEK1* | | NIMA (never in mitosis gene a)-related kinase 1 |
|  | | *HIF1A*  *DHX16*  *SNX4*  *MYF6*  *TBC1D23* | | Hypoxia-inducible factor 1, alpha subunit (basic helix-loop-helix transcription factor) |
|  | |  |  | DEAH (Asp-Glu-Ala-His) box polypeptide 16 |
|  | |  |  | Sorting nexin 4 |
|  | |  |  | Myogenic factor 6 (herculin) |
|  | |  |  | TBC1 domain family, member 23 |
| Up-regulated  E treatment | | *LFNG* | | O-fucosylpeptide 3-beta-N-acetylglucosaminyltransferase |
|  | | *SEPT7* | | Septin 7 |
|  | | *C19orf53* | | Chromosome 19 open reading frame 53 |
|  | | *FRS3* | | Fibroblast growth factor receptor substrate 3 |
|  | | *HNRPK* | | Heterogeneous nuclear ribonucleoprotein K |
|  | | *CDH4* | | Cadherin 4, type 1, R-cadherin (retinal) |
|  | | *LRP6* | | Low density lipoprotein receptor-related protein 6 |
|  | | *APH1B* | | Anterior pharynx defective 1 homolog B (C. elegans) |
|  | | *RAB2B* | | RAB2B, member RAS oncogene family |
|  | | *ELOF1* | | Elongation factor 1 homolog (S. cerevisiae) |
|  | | *FAM124B* | | Family with sequence similarity 124B |
|  | | *GDPD3* | | Glycerophosphodiester phosphodiesterase domain containing 3 |
|  | | *FAT* | | FAT tumor suppressor homolog 1 (Drosophila) |
|  | | *TSEN34* | | TRNA splicing endonuclease 34 homolog (S. cerevisiae) |
|  | | *SUPT3H* | | Suppressor of Ty 3 homolog (S. cerevisiae) |
|  | | *ANAPC11* | | APC11 anaphase promoting complex subunit 11 homolog (yeast) |
|  | | *HERC6* | | Hect domain and RLD 6 |
|  | | *NFKB2* | | Nuclear factor of kappa light polypeptide gene enhancer in B-cells 2 (p49/p100) |
|  | | *RBPMS* | | RNA binding protein with multiple splicing |
|  | | *C1orf51* | | Chromosome 1 open reading frame 51 |
|  | | *SEMA6C* | | Sema domain, transmembrane domain (TM), and cytoplasmic domain, (semaphorin) 6C |
|  | | *EFS* | | Embryonal Fyn-associated substrate |
|  | | *RPS6KB2* | | Ribosomal protein S6 kinase, 70kDa, polypeptide 2 |
|  | | *CBLL1* | | Cas-Br-M (murine) ecotropic retroviral transforming sequence-like 1 |
|  | | *PGBD5* | | PiggyBac transposable element derived 5 |
|  | | *SPINK1* | | Serine peptidase inhibitor, Kazal type 1 |
|  | | *KIAA0427* | | KIAA0427 |
|  | | *CFLAR* | | CASP8 and FADD-like apoptosis regulator |
|  | | *FAM70A* | | Family with sequence similarity 70, member A |
|  | | *SPRR1B* | | Small proline-rich protein 1B (cornifin) |
|  | | *INTS7* | | Integrator complex subunit 7 |
|  | | *ZMIZ2* | | Zinc finger, MIZ-type containing 2 |
|  | | *CTA-216E10.6* | | Hypothetical protein FLJ23584 |
|  | | *DEFB119* | | Defensin, beta 119 |
|  | | *YIF1A* | | Yip1 interacting factor homolog A (S. cerevisiae) |
|  | | *SLC29A2* | | Solute carrier family 29 (nucleoside transporters), member 2 |
|  | | *CABIN1* | | Calcineurin binding protein 1 |
|  | | *LBP* | | Lipopolysaccharide binding protein |
|  | | *C19orf56* | | Chromosome 19 open reading frame 56 |
|  | | *OTUB2* | | OTU domain, ubiquitin aldehyde binding 2 |
|  | | *KRT32* | | Keratin 32 |
|  | | *SCO1* | | SCO cytochrome oxidase deficient homolog 1 (yeast) |
|  | | *RAB40B* | | RAB40B, member RAS oncogene family |
|  | | *ASXL2* | | Additional sex combs like 2 (Drosophila) |
|  | | *KCNG2* | | Potassium voltage-gated channel, subfamily G, member 2 |
|  | | *PJA1* | | Praja 1 |
|  | | *TXNL5* | | Thioredoxin-like 5 |
|  | | *BTN2A3* | | Butyrophilin, subfamily 2, member A3 |
|  | | *RAD9A* | | RAD9 homolog A (S. pombe) |
|  | | *ABCC10* | | ATP-binding cassette, sub-family C (CFTR/MRP), member 10 |
|  | | *TUSC2* | | Tumor suppressor candidate 2 |
|  | | *TCP10L* | | T-complex 10 (mouse)-like |
|  | | *RPP21* | | Ribonuclease P 21kDa subunit |
|  | | *CRYAA* | | Crystallin, alpha A |
|  | | *FKBP1A* | | FK506 binding protein 1A, 12kDa |
|  | | *LRRC8A* | | Leucine rich repeat containing 8 family, member A |
|  | | *ATP5G1* | | ATP synthase, H+ transporting, mitochondrial F0 complex, subunit C1 (subunit 9) |
|  | | *HPS6* | | Hermansky-Pudlak syndrome 6 |
|  | | *CDC42EP4* | | CDC42 effector protein (Rho GTPase binding) 4 |
|  | | *DDX54* | | DEAD (Asp-Glu-Ala-Asp) box polypeptide 54 |
|  | | *AP2S1* | | Adaptor-related protein complex 2, sigma 1 subunit |
|  | | *JARID2* | | Jumonji, AT rich interactive domain 2 |
|  | | *RPS23* | | Ribosomal protein S23 |
|  | | *MTCH2* | | Mitochondrial carrier homolog 2 (C. elegans) |
|  | | *CUTA* | | CutA divalent cation tolerance homolog (E. coli) |
|  | | *PNPLA5* | | Patatin-like phospholipase domain containing 5 |
|  | | *MARCH5* | | Membrane-associated ring finger (C3HC4) 5 |
|  | | *TRIM47* | | Tripartite motif-containing 47 |
|  | | *HMCN1* | | Hemicentin 1 |
|  | | *SLC12A7* | | Solute carrier family 12 (potassium/chloride transporters), member 7 |
|  | | *BCL7C* | | B-cell CLL/lymphoma 7C |
|  | | *PLXNB1* | | Plexin B1 |
|  | | *DLGAP3* | | Discs, large (Drosophila) homolog-associated protein 3 |
|  | | *WDR67* | | WD repeat domain 67 |
|  | | *CENTD3* | | Centaurin, delta 3 |
|  | | *TUB* | | Tubby homolog (mouse) |
|  | | *IGF2AS* | | Insulin-like growth factor 2 antisense |
|  | | *SERF2* | | Small EDRK-rich factor 2 |
|  | | *PITPNM1* | | Phosphatidylinositol transfer protein, membrane-associated 1 |
|  | | *PPAN* | | Peter pan homolog (Drosophila) |
|  | | *FAM55D* | | Family with sequence similarity 55, member D |
|  | | *SNRPB* | | Small nuclear ribonucleoprotein polypeptides B and B1 |
|  | | *NRIP2* | | Nuclear receptor interacting protein 2 |
|  | | *IGSF9B* | | Immunoglobulin superfamily, member 9B |
|  | | *FNDC3B* | | Fibronectin type III domain containing 3B |
|  | | *GDF10* | | Growth differentiation factor 10 |
|  | | *GSTO1* | | Glutathione S-transferase omega 1 |
|  | | *YIPF3* | | Yip1 domain family, member 3 |
|  | | *ATP11A* | | ATPase, Class VI, type 11A |
|  | | *POLR1E* | | Polymerase (RNA) I polypeptide E, 53kDa |
|  | | *PLEKHA5* | | Pleckstrin homology domain containing, family A member 5 |
|  | | *GPR88* | | G protein-coupled receptor 88 |
|  | | *PTCHD2* | | Patched domain containing 2 |
|  | | *MTA1* | | Metastasis associated 1 |

Genes were selected with p-values < 0.05 and a fold change above 2 (Up-regulated) or below ½(Down-regulated) in both cell lines. E = Epidermal growth factor 10 nmol/L; Cx10 = cetuximab 10 nmol/L; Gb = gefitinib 1μmol/L.
